# Adaptive radiation along a deeply conserved genetic line of least resistance in *Anolis* lizards

**DOI:** 10.1002/evl3.72

**Published:** 2018-07-17

**Authors:** Joel W. McGlothlin, Megan E. Kobiela, Helen V. Wright, D. Luke Mahler, Jason J. Kolbe, Jonathan B. Losos, Edmund D. Brodie

**Affiliations:** ^1^ Department of Biological Sciences Virginia Tech Blacksburg Virginia 24061; ^2^ Department of Ecology Evolution, and Behavior, University of Minnesota St. Paul Minnesota 55108; ^3^ Computing Community Consortium Computing Research Association Washington District of Columbia 20036; ^4^ Department of Ecology and Evolutionary Biology University of Toronto Toronto Ontario M5S 3B2 Canada; ^5^ Department of Biological Sciences University of Rhode Island Kingston Rhode Island 02881; ^6^ Department of Biology Washington University Saint Louis Missouri 63130; ^7^ Department of Biology and Mountain Lake Biological Station University of Virginia Charlottesville Virginia 22904

**Keywords:** Adaptive radiation, *Anolis* lizards, constraint, convergent evolution, covariance tensor analysis, G matrix, quantitative genetics, selection

## Abstract

On microevolutionary timescales, adaptive evolution depends upon both natural selection and the underlying genetic architecture of traits under selection, which may constrain evolutionary outcomes. Whether such genetic constraints shape phenotypic diversity over macroevolutionary timescales is more controversial, however. One key prediction is that genetic constraints should bias the early stages of species divergence along “genetic lines of least resistance” defined by the genetic (co)variance matrix, G. This bias is expected to erode over time as species means and G matrices diverge, allowing phenotypes to evolve away from the major axis of variation. We tested for evidence of this signal in West Indian *Anolis* lizards, an iconic example of adaptive radiation. We found that the major axis of morphological evolution was well aligned with a major axis of genetic variance shared by all species despite separation times of 20–40 million years, suggesting that divergence occurred along a conserved genetic line of least resistance. Further, this signal persisted even as G itself evolved, apparently because the largest evolutionary changes in G were themselves aligned with the line of genetic least resistance. Our results demonstrate that the signature of genetic constraint may persist over much longer timescales than previously appreciated, even in the presence of evolving genetic architecture. This pattern may have arisen either because pervasive constraints have biased the course of adaptive evolution or because the G matrix itself has been shaped by selection to conform to the adaptive landscape.

**Impact summary**

Evolutionary biologists have long debated whether biodiversity is shaped mainly by natural selection or by intrinsic factors, such as genetic variation and the developmental mechanisms that translate genes into phenotype. The importance of selection has been convincingly demonstrated many times, but the extent to which genetic architecture might constrain the long‐term outcomes of selection is poorly understood. In this study, we use the adaptive radiation of *Anolis* lizards in the West Indies to show that genetic architecture aligns with phenotypic change for up to 40 million years, about ten times longer than previously predicted. We show that this signature is even maintained when the genetic constraints themselves evolve. Although the pattern we demonstrate is consistent with genetic constraints biasing evolutionary change, it is equally consistent with the action of natural selection simultaneously shaping traits and the genetic variation that underlies them. Depending on what mechanisms are ultimately responsible for these patterns, our results may have one of two equally exciting implications. On the one hand, elaborate adaptive radiations like the one seen in West Indian anoles may be possible even when genetic constraints persist for millions of years. On the other hand, genetic constraints may respond to natural selection in such a way as to facilitate further adaptive evolution.

Both natural selection and genetic architecture play important roles in determining the direction and magnitude of evolutionary change (Lande 1976, 1979). On the scale of a few generations, the interactions between these factors are well understood. Adaptive evolution proceeds when natural selection favors change, and genetic architecture (i.e., the patterns of genetic variation and covariation underlying trait expression) determines whether and how traits respond to selection across generations (Lande 1979; Grant and Grant 1995). In the short run, features of genetic architecture such as limited genetic variation or strong genetic correlations may lead to constraints that bias evolutionary response to selection toward certain directions, while slowing or prohibiting evolution in other directions (Arnold 1992; Blows and Hoffmann 2005; Walsh and Blows 2009). However, the extent to which genetic constraints influence larger scale evolutionary change, such as phenotypic divergence in species radiations, remains a major unresolved question in biology (Schluter 2000; Gould 2002).

In the early stages of species divergence, evolution is predicted to be biased along “genetic lines of least resistance” defined by **G**, the additive genetic variance‐covariance matrix (Schluter 1996; McGuigan 2006). A number of studies have provided empirical support for this prediction, but most work has been conducted on relatively short evolutionary timescales (1–2 million years, Schluter 1996; Blows and Higgie 2003; Bégin and Roff 2004; McGuigan et al. 2005; Hansen and Houle 2008; Chenoweth et al. 2010; Bolstad et al. 2014; Walter et al. 2018). Genetic constraints are often considered to be less important over the longer evolutionary spans that generate species differences, but there are few empirical tests of this prediction (but see Houle et al. 2017). One reason constraint might be less of a factor on macroevolutionary timescales is that **G** itself can evolve (Turelli 1988; Steppan et al. 2002; Arnold et al. 2008), potentially altering the genetic lines of least resistance to reflect the adaptive landscape (Arnold et al. 2001). Both theoretical (Lande 1980; Jones et al. 2003; Arnold et al. 2008) and empirical results (Steppan et al. 2002; Roff and Fairbairn 2012; Björklund et al. 2013; Careau et al. 2015) indicate that selection and drift can alter the characteristics of **G**, but it is unknown whether such changes tend to preserve or alter genetic lines of least resistance (but see Walter et al. 2018).

Here, we use a comparative study of *Anolis* lizards to assess the relationship between genetic constraints and phenotypic divergence in adaptive radiation. In the West Indies, anoles have repeatedly diversified, with a similar set of habitat specialist types, known as ecomorphs, evolving independently on different islands (Williams 1972; Losos et al. 1998; Losos 2009; Mahler et al. 2013). Among other traits, ecomorphs differ notably in relative limb length, which allows different ecomorphs to perform well in different microhabitats. Here, we focus primarily on two ecomorphs, trunk‐ground and trunk‐crown, which respectively have relatively long and relatively short limbs suitable for locomotion on different types of perches (Losos 1990a; Losos and Irschick 1996; Irschick and Losos 1998). We also include one representative of a third ecomorph, grass‐bush, which has a narrow body and relatively long hindlimbs. The role of natural selection in the repeated evolution of ecomorph‐specific traits, which is supported by a large body of evidence (Losos 2009), suggests that phenotypic divergence in anoles is unlikely to have been limited by genetic architecture. In addition, the age of the *Anolis* radiation (46.3–64.4 million years, Poe et al. 2017) indicates that there has likely been ample time both for diverging species to approach their evolutionary optima and for **G** matrices to diverge in response to selection or drift. Both these considerations suggest that morphological divergence is unlikely to be aligned with genetic lines of least resistance.

We take a multivariate approach to dissecting patterns of genetic architecture and their relationships with phenotypic divergence among seven *Anolis* species from three different island lineages. We use animal models to estimate both species‐specific genetic architecture (**G** matrices) for a suite of skeletal traits and the direction and magnitude of evolutionary divergence among *Anolis* species in size‐corrected morphological space. We then explore whether the major axes of genetic variation for each species share orientation in multivariate space (Krzanowski common subspace analysis). We find that two axes describe the majority of genetic variation in all seven species, and that these directions are aligned to the major axis of genetic variation in an ancestral **G** matrix reconstructed to represent the hypothetical pattern of ancestral genetic architecture. To ask whether **G** itself evolved during the adaptive radiation, we analyze covariance tensors and find that most differentiation of genetic architecture occurs in subspaces that include limb traits. By comparing angles of orientation of these major axes of phenotypic divergence (**d**), genetic variation (**h**), and genetic differentiation (**e**), we reveal that both trait means and genetic covariance structure appear to evolve most rapidly along lines of genetic least resistance.

## Methods

### ESTIMATION OF G

**Figure 1:**
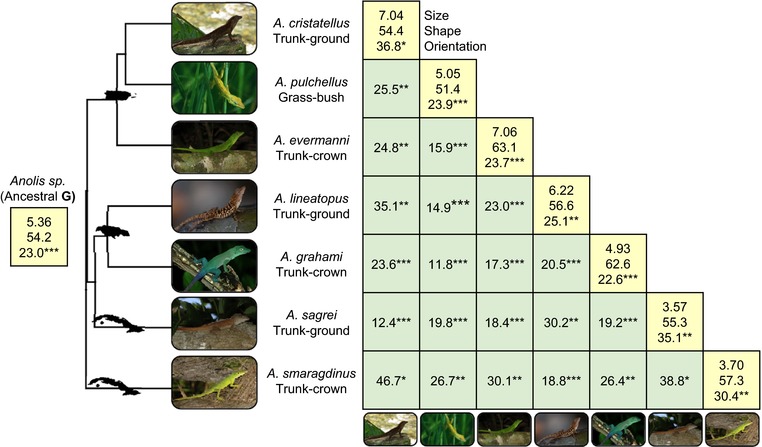
Divergence of genetic architecture across the *Anolis* radiation. Numbers on the diagonal represent size (total genetic variance × 10^3^), shape (percent variance explained by g_max_), and orientation (vector angle between g_max_ and the major axis of divergence, d_1_). Numbers off the diagonal represent the angle in degrees between g_max_ vectors for a species pair. All estimates of g_max_ were significantly more aligned than expected by chance (see Methods; ^*^ *P* < 0.05, ^**^ *P* < 0.01, ^***^ *P* < 0.001). All estimates of g_max_ were also aligned with both d_1_ and h_1_, the axis of greatest shared genetic variance (Table S4). Statistics for a reconstruction of the ancestral G using maximum likelihood are presented at the root of the phylogeny (Zheng and Wiens 2016), which has an estimated date of 41.5–43.5 million years ago (Zheng and Wiens 2016; Poe et al. 2017). The most recent splits in the phylogeny occurred approximately 19.8–22.5 million years ago (Zheng and Wiens 2016; Poe et al. 2017). The island of origin for each group is indicated on the phylogeny (from top to bottom, Puerto Rico, Jamaica, and Cuba).

In a common laboratory environment, we estimated **G** matrices for seven species of West Indian *Anolis* lizards representing three different ecomorphs (trunk‐crown, trunk‐ground, and grass‐bush) that originated independently on three different islands of the Greater Antilles: *A. cristatellus* (trunk‐ground), *A. evermanni* (trunk‐crown), and *A. pulchellus* (grass‐bush) from Puerto Rico; *A. grahami* (trunk‐crown) and *A. lineatopus* (trunk‐ground) from Jamaica; and *A. sagrei* (trunk‐ground) and *A. smaragdinus* (trunk‐crown) from South Bimini, the Bahamas (Table 1). Both Bahamian species are from lineages that originated in Cuba and colonized the Bahamas naturally (Kolbe et al. 2004; Glor et al. 2005). These species represent lineages separated by approximately 20–40 million years (Fig. 1) (Zheng and Wiens 2016; Poe et al. 2017).

**Table 1:** Study design

Species name	Ecomorph	Island of collection	Coordinates	Sires	Dams	Juveniles
*A. cristatellus*	Trunk‐ground	Puerto Rico	18.05°N, 65.83°W	67	109	643
*A. pulchellus*	Grass‐bush	Puerto Rico	18.26°N, 65.71°W	35	62	430
*A. evermanni*	Trunk‐crown	Puerto Rico	18.27°N, 65.72°W	68	105	469
*A. lineatopus*	Trunk‐ground	Jamaica	18.32°N, 76.81°W	30	42	259
*A. grahami*	Trunk‐crown	Jamaica	18.32°N, 76.81°W	32	35	144
*A. sagrei*	Trunk‐ground	South Bimini, Bahamas (Cuban lineage)	25.70°N, 79.28°W	55	99	791
*A. smaragdinus*	Trunk‐crown	South Bimini, Bahamas (Cuban lineage)	25.70°N, 79.28°W	43	60	168

We used a half‐sibling breeding design to estimate **G** matrices for a suite of eight skeletal traits: jaw length, head width, pectoral width, pelvic width, humerus length, ulna length, femur length, and tibia length. Across all seven species, we measured 9369 individual X‐ray images from 2904 lab‐reared juveniles from 512 maternal families (Table 1). Traits were measured from X‐ray images of juveniles taken at four points during development, and **G** matrices were estimated ASReml (Gilmour et al. 2009) using multivariate repeated‐measures animal models of natural‐log transformed traits with natural‐log snout‐vent length (SVL) as a covariate. Details of collection, husbandry and breeding, phenotyping, and quantitative genetic analyses are given in Additional Methods (Supporting Information). Symbols used in this article are listed in Table 2.

**Table 2:** Symbols used in this article

Symbol	Definition
**G**	The additive genetic variance‐covariance matrix
**G**_anc_	The ancestral **G** matrix, estimated using maximum likelihood.
**g**_max_	The largest eigenvector of **G**; describes the combination of traits that represent the most genetic variance.
**D**	The among‐species divergence matrix; describes the phenotypic differentiation among the species in the study, calculated as a variance‐covariance matrix of species means.
**d** *_i_*	The eigenvectors of **D**; the largest eigenvector, **d**_1_, describes the combination of traits with that captures the most divergence among taxa.
**H**	The common subspace of genetic variation for all seven species; describes the orientations of trait space that share the most genetic variation and is defined using the first four eigenvectors of each **G** matrix.
**h** *_i_*	The eigenvectors of **H**; **h**_1_ is an analog of **g**_max_ that describes the major axes of genetic variance shared across species.
**E** *_i_*	The eigentensors describing subspaces in which **G** varies across species.
**e** *_ij_*	The *j*th eigenvector of the *i*th eigentensor; describes trait combinations for which genetic variance has diverged among all species.
**M**	The variance‐covariance matrix of per‐generation mutational input.
θ	The vector angle, given in degrees.
*G*	Genetic variance explained by a given eigenvector in a common subspace.
*D*	Divergence explained by a given eigenvector in a common subspace.
*D_G_*	Divergence in **G** explained by a given eigenvector in a common subspace.

### SPECIES DIVERGENCE

Parameters from our animal models were used to quantify species divergence in morphology. Using estimated intercepts, slopes from the regression of ln‐transformed trait values on ln‐transformed SVL, and the grand mean SVL across all seven species (34.81 mm; Table S2), we calculated size‐corrected species means for each trait. This approach allowed us to determine how species had diverged in shape while controlling for species differences in overall size. We performed eigenanalysis of the variance‐covariance matrix of species means (**D**) to determine axes of greatest divergence (eigenvectors, **d**_1_‐**d**_6_) and the variance explained by each (eigenvalues). As described in Additional Methods, we also calculated two alternative estimates of species divergence that accounted for phylogeny and a third from a separate dataset of measurements from wild‐collected adult males of 15 species.

### ANALYSIS OF G MATRICES

#### Descriptive statistics and visualization

We performed eigenanalysis (generating eight eigenvectors, **g**_max_ and **g**_2_‐**g**_8_) for each **G** matrix and calculated several descriptive statistics to aid in the interpretation of their overall structure. The trace, or the sum of the eigenvalues of each **G** matrix (which is equivalent to the sum of the genetic variances), was used as an index of its overall size, which should predict the potential magnitude of a population's overall response to selection. The percent variance explained by **g**_max_ (the axis of greatest additive genetic variance) was used as an index of **G** matrix shape, which indicates a population's potential to respond to selection aligned with **g**_max_ relative to other directions. Finally, we calculated the angle between **g**_max_ estimates from each species and the vector of greatest species divergence (**d**_1_) as an index of orientation. As an additional measure of orientation, we calculated all pairwise angles between species‐specific estimates of **g**_max_. These values indicate the degree to which **G** matrices vary in the direction of greatest genetic variation.

**Figure 2:**
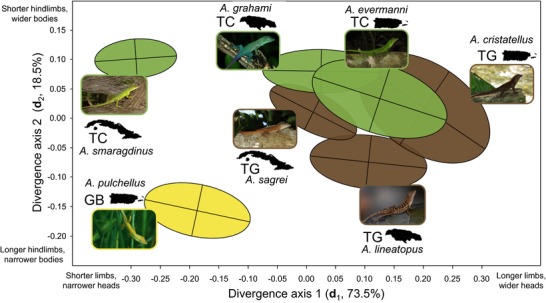
Relationship between species divergence and genetic architecture. Species‐specific G matrices were visualized by transforming estimated breeding values for each trait using the divergence eigenvectors d_1_ and d_2_ and plotting 95% confidence ellipses centered at the multivariate species mean. Ellipses are color‐coded by ecomorph, with trunk‐crown (TC) species in green, trunk‐ground (TG) species in brown, and the grass‐bush (GB) species in yellow. The major axis of morphological divergence (d_1_) is aligned with the major axis of genetic variance shared by all G matrices (h_1_; Table 4). The axis of greatest divergence in G (e_11_) is aligned with d_1_ and primarily represents changes in G‐matrix size (total genetic variance; Figs. 1, 3, Table 4). See Fig. 1 for island names.

**Figure 3:**
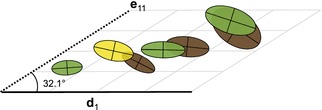
Representations of G matrices as ellipses (as in Fig. 2), plotted by species coordinates within the axes of greatest divergence of species means (d_1_) and the subspace with the greatest divergence of G matrices (E_1_, represented by its first eigenvector e_11_). Ellipses to the right are larger, illustrating the correlation between matrix size (total genetic variance) and species coordinates within E_1_.

To visualize **G** matrices in two dimensions, we estimated best‐linear unbiased predictors of breeding values for each trait in ASReml and transformed them using the coefficients of **d**_1_ and **d**_2_, the axes of greatest morphological divergence. We then plotted the 95% confidence ellipse centered at the species mean using JMP Pro 13.0. Although these plots were not used for any formal analyses, they facilitate visual comparison of **G**‐matrix size, shape, and orientation (see Figs. 2 and 3).

#### Detection of similar axes of genetic variation

After estimating **G** matrices, we conducted analyses allowing us to characterize both similarities and differences across species. Matrices with different eigenstructure may still have axes of genetic variation pointing in similar directions in trait space. Such similarities can be characterized using Krzanowski's common subspace analysis (Krzanowski 1979; Aguirre et al. 2014; Melo et al. 2015), which calculates the subspace (**H**) describing the greatest similarity across a set of matrices. Eigenanalysis of this subspace provides a set of orthogonal vectors (**h** *_i_*) that represent axes of genetic variance that are shared to some extent across species, and its eigenvalues (*p*) indicate the extent to which those axes are shared. In our analyses, these eigenvalues could range from 0 to 7, the number of species. An eigenvalue of 7 would indicate that a particular **h** vector can be reconstructed exactly for all seven species using the eigenvectors of its **G** matrix, and would suggest that a given eigenvector represented a conserved axis of genetic variance.

We calculated subspace **H** using the first four eigenvectors of each **G** matrix, the maximum allowed for an 8 × 8 matrix (Aguirre et al. 2014) and performed eigenanalysis to estimate four vectors, **h**_1_–**h**_4_, and their associated eigenvalues (Table S4). Next, we calculated the angles between each **h** vector and the subspace defined by the first four eigenvectors of **G** within each species (Aguirre et al. 2014). The closer these angles are to 0°, the better a particular **h** vector describes genetic variance within a particular species. If a particular **h** vector is aligned with all **G** matrices, this would further indicate that it describes an axis of genetic variance that is conserved across species. We also calculated the amount of species‐specific genetic variance explained by each **h** vector by projecting it through each **G** matrix using the equation **h** ^T^ **Gh**, where T denotes transposition (Aguirre et al. 2014). Finally, to assess the degree to which the eigenvectors of **H** were similar to those of **G** for each species, we calculated the angle between each **h** vector and the corresponding eigenvector of **G** (i.e., **h**_1_ vs **g**_max_, **h**_2_ vs **g**_2_, etc.).

As an additional way to explore conserved axes of genetic variance, we reconstructed an ancestral **G** matrix (**G**_anc_) using element‐by‐element maximum likelihood reconstruction, a time‐calibrated phylogeny (pruned from Zheng and Wiens 2016), and a Brownian motion model of evolution in APE (Paradis et al. 2004) (Table S1). These analyses must be interpreted with caution because **G‐**matrix evolution likely does not conform to a Brownian motion model (Liam Revell, pers. commun.); however, they do offer the advantage of incorporating phylogenetic structure, which cannot be accomplished using Krzanowski's method. The eigenvectors of **G**_anc_ were highly similar to those of **H**, indicating that they described a similar subspace. Substituting these eigenvectors for **h** *_i_* in our subsequent analyses did not change our results. To visualize species differences in multivariate genetic variance, we projected each of these eigenvectors through each species‐specific **G** matrix to calculate genetic variance in a common set of orthogonal trait combinations.

#### Patterns of **G**‐matrix divergence

We used genetic covariance tensor analysis (Hine et al. 2009; Aguirre et al. 2014) to characterize the directions in which **G** diverged across species (Table S2). This analysis allowed us to determine directions in trait space with the largest changes in genetic variance across species.

The genetic covariance tensor is a fourth‐order analog of a variance‐covariance matrix that describes among‐species variation in **G**, its elements describing (co)variances of (co)variances. Eigenanalysis of this tensor provides genetic covariance eigentensors (**E** *_i_*), which are square matrices describing independent subspaces in which in **G** varies across species. Analogous to a first principal component, the first eigentensor describes the subspace in which **G** varies the most across species. The coordinates of each species within each eigentensor can be calculated to determine the extent to which species differ in a particular subspace (Hine et al. 2009). Each eigentensor can also be further decomposed into eigenvectors (**e** *_ij_*, denoting the *j*th eigenvector of the *i*th eigentensor), which describe linear combinations of the original traits that contribute to divergence in **G**. Within each eigentensor, an eigenvector associated with the eigenvalue of the largest absolute value describes the combination of traits whose genetic variance differs the most across species. We used the method described by Hine et al. (2009) to calculate the proportion of total divergence in **G** explained by each eigenvector **e** *_ij,_* which is a function of the eigenvalues of both the eigenvector itself and its associated eigentensor. Because eigenvalues may be negative, the eighth eigenvector within an eigentensor sometimes explains a large amount of divergence in **G** (Table S5).

### RELATIONSHIPS BETWEEN G AND DIVERGENCE

Determining the relationship between evolutionary divergence and **G** is difficult when **G** does not remain constant across diverging taxa. Most tests of evolution along genetic lines of least resistance follow Schluter (1996), comparing divergence of species means to a single estimate of **G**. We used a different approach that allows us to capture information from all of estimates of **G** within the radiation. First, we compared the orientation of the axes of greatest phenotypic divergence (**d** *_i_*) to that of conserved axes of genetic variance identified via Krzanowski's common subspace analysis (**h** *_i_*). Because these vectors describe axes of genetic variance across all species, they represent putative genetic lines of least resistance that may have influenced divergence. Alignment of **d** and **h** vectors would indicate that evolutionary change was biased toward such lines of least resistance. Second, we asked whether the divergence of **G** itself occurred in directions predicted by either morphological divergence or conserved axes of genetic variance by calculating the angles between the largest ten eigenvectors (**e** *_ij_*) from the tensor analysis and **d** *_i_* and **h** *_i_* vectors, respectively. Alignment of **e** *_ij_* with **d** *_i_* would suggest that **G**‐matrix evolution was influenced by the same factors that led to divergence in species means. Alignment of **e** *_ij_* with **h** *_i_* would show that divergence in **G** occurred in directions similar to conserved axes of genetic variation, suggesting that evolution of **G** is itself subject to constraints, or alternatively, that both standing genetic variation and divergence of **G** across species were influenced in a similar way by a third factor, such as selection or drift.

To perform each of these comparisons, we calculated angles (θ) between different types of vectors (**d** *_i_*, **h** *_i_*, and **e** *_ij_*), which may range from 0° (completely aligned) to 90° (orthogonal). All tests involving **d** vectors were repeated using our alternative measures of species divergence (see “Species Divergence” above and Additional Methods). Because the direction of eigenvectors is arbitrary, we reversed the sign of one of the vectors if the calculated angle was above 90°. To determine whether vectors were significantly aligned, we compared this angle to a null distribution generated from a simulation of 100,000 pairs of randomly generated vectors. We constructed each random vector by drawing its eight elements from a uniform distribution bounded by –1 and 1 and then standardizing the vector to unit length. The critical values from this null distribution were 47.6° (*P* = 0.05), 35.7° (*P* = 0.01), and 24.0° (*P* = 0.001).

As an additional test for the relationship between **G** and divergence of species means, we asked whether trait combinations with more genetic variance consistently showed greater divergence following the method of Houle et al. (2017). First, we scaled the estimated ancestral **G** matrix to the same size as **D** by multiplying it by a correction factor (the trace of **D** divided by the trace of **G**_anc_). Then we calculated the average of the rescaled **G**_anc_ and **D** and calculated the eigenvectors of the resultant matrix, providing a set of orthogonal vectors representing a subspace common to **G**_anc_ and **D**. Next, these eigenvectors were projected through both of the original matrices to determine the amount of within‐species genetic variance and among‐species variance, respectively, for each trait combination. We then regressed log_10_‐transformed among‐species variances (log *D*) on log_10_‐transformed genetic variances (log *G*). A positive relationship would indicate an association between greater genetic variance and divergence, and the slope of this regression represents the scaling parameter for the relationship between **G**_anc_ and **D**, the predicted value of which varies across different models of evolution (Houle et al. 2017). This test was repeated using two other measures of species divergence as well as the evolutionary rate matrix (see Additional Methods).

Analogously, to ask how change in **G** for a particular trait combination scaled with available genetic variation, we performed a similar regression of log_10_‐transformed among‐species divergence in genetic variance (log *D_G_*) on log *G*. For the latter analysis, we used species‐specific genetic variances in the eigenvectors of **G**_anc_ to calculate log *E* and the eigenvalues of **G**_anc_ to calculate log *G*.

## Results

### CHARACTERISTICS OF G

Of the three metrics we used to characterize **G** matrices, size (total genetic variance) varied the most across species (coefficient of variation = 27%), followed by orientation (angle of **g**_max_, 21%), and shape (% variance explained by **g**_max_, 7.5%; Fig. 1). In all species, the axis of greatest genetic variance (**g**_max_) was strongly associated with genetic variance in limb traits, which consistently showed strong positive loadings (Table S1). Across species, all **g**_max_ vectors were significantly aligned with one another (θ = 11.8–46.7°, *P* < 0.05), but none were collinear (Fig. 1).

### MORPHOLOGICAL DIVERGENCE

The major axis of morphological divergence (**d**_1_, explaining 73.5% of divergence) separated species with long limbs and wide heads from those with shorter limbs and narrow heads (Fig. 3; Tables 3, S2). This axis separated trunk‐crown from trunk‐ground species within islands and separated the grass‐bush species *A*. *pulchellus* from the other Puerto Rican species. In addition, within each island, trunk‐ground species had slightly higher scores for **d**_1_ than did trunk‐crown species. The second axis of divergence (**d**_2_, 18.5%) separated species with wider bodies and relatively short hindlimbs from those with narrow bodies and longer hindlimbs (Fig. 2; Tables 3, S2). This axis further separated all three ecomorphs, as trunk‐crown species have short hindlimbs and wide bodies, trunk‐ground species have long hindlimbs and slightly narrower bodies, and the grass‐bush species has long hindlimbs and a very narrow body. Alternative estimates of divergence (see Additional Methods) had similar eigenstructure to **D** (Tables S2, S3; Fig. S2). These patterns are consistent with previous analyses of divergence in the West Indian *Anolis* radiation and reflect both divergence among islands and habitat specialization within lineages (Losos et al. 1998; Beuttell and Losos 1999; Losos 2009; Mahler et al. 2013).

**Table 3:** Eigenvectors of conserved genetic variation (**h**), divergence in means (**d**), and divergence in **G** (**e**)

	d_1_	d_2_	h_1_	h_2_	e_11_	e_28_
% variance	73.5	18.5	46.4–60.8	13.0–29.6	40.7	12.7
Jaw length	0.017	0.035	0.187	0.185	–0.020	–0.349
Head width	0.365	0.369	0.139	0.391	0.006	–0.182
Pectoral width	0.140	0.424	0.250	0.756	–0.104	–0.512
Pelvic width	0.216	0.511	0.197	0.320	–0.019	–0.315
Humerus	0.323	0.209	0.500	–0.180	0.459	–0.110
Ulna	0.532	0.039	0.491	–0.186	0.411	–0.216
Femur	0.373	–0.375	0.401	–0.172	0.531	–0.512
Tibia	0.522	–0.485	0.438	–0.207	0.571	–0.403

For **h**, eigenvectors derive from Krzanowski's common subspace analysis (Table S4) and percent variance is given as a range when **h** is projected through species‐specific **G** matrices. For **e**, eigenvectors derive from covariance tensor analysis (Table S5) and percent variance is the amount of divergence in **G** explained. For **e** vectors, subscripts refer to the subspace (**E**_1_–**E**_6_) and the vector number within the subspace (1–8).

### IDENTIFICATION OF GENETIC LINES OF LEAST RESISTANCE

Despite the divergence of **G** across species, two axes of genetic variation identified by Krzanowski's common subspace analysis, **h**_1_ and **h**_2_, adequately described the majority of genetic variation across all species (*P* = 6.94 and 6.56, respectively, out of a possible 7; Tables 3, S4). The first of these axes (**h**_1_) explained between 46 and 61% of genetic variance within each species and was similar (but not identical) to each species‐specific **g**_max_ (Table S4) as well as to an ancestral reconstruction of **g**_max_ (θ = 10.7°). These patterns suggest that **h**_1_ represents a conserved genetic line of least resistance. Like **g**_max_, this axis was most strongly loaded with limb traits. A second axis (**h**_2_) explained between 13 and 30% of genetic variance within species (Tables 3, S4). This axis primarily described genetic variance in body and head width. Two other axes (**h**_3_ and **h**_4_) were less similar across species (*P* = 5.89 and 4.32, respectively) and captured a smaller amount of genetic variance within each species (5–14% and 3–15%, respectively). Together, **h**_1_ **–h**_4_ captured between 83% and 94% of total genetic variance within species.

### PATTERNS OF G‐MATRIX DIVERGENCE

Genetic covariance tensor analysis showed that 84% of divergence in **G** could be explained by the first three of six independent subspaces (**E**_1_–**E**_3_). Species coordinates in the first eigentensor (**E**_1_), which explained 48% of divergence in **G**, were highly correlated with the trace of **G** (total genetic variance), suggesting that the largest changes in **G** were changes in size (Fig. 3, Table S5; *r* = 0.95, *P* = 0.001). A single combination of traits (**e**_11_) within **E**_1_ was responsible for 41% of the overall divergence in **G** (Table 3). Examination of the loadings of **e**_11_ indicates that it almost entirely represents divergence in the components of **G** involving limb length. Species coordinates within the second eigentensor (**E**_2_) were marginally correlated with the orientation of **G** (Table S5; *r* = 0.73, *P* = 0.06).

### RELATIONSHIPS BETWEEN G AND DIVERGENCE

The major axis of divergence (**d**_1_) was closely aligned with the major axis of conserved genetic variance (**h**_1_; Table 4), suggesting that a majority of phenotypic divergence has occurred along the genetic line of least resistance. The second axis of divergence (**d**_2_) was nearly orthogonal to **h**_1_ (Table 4) and was significantly but weakly aligned with the next axis of available genetic variation (**h**_2_; Table 4). The relationship between divergence and genetic variance can also be seen by examining the orientation of each individual **G** matrix, as the **g**_max_ of each species was significantly aligned with **d**_1_ (θ = 23–37°; Fig. 1). This pattern is visualized in Fig. 2, where **G** matrices are plotted as ellipses in the subspace defined by **d**_1_ and **d**_2_ and centered on species means. Here, the axis capturing the most genetic variance in this subspace—the major axis of each ellipse—tends to be biased toward **d**_1_.

**Table 4:** Angles between vectors in Table 3, given in degrees

	**d**_1_	**d**_2_	**h**_1_	**h**_2_
**h**_1_	21.0***	88.6		
**h**_2_	89.5	41.0*		
**e**_11_	32.1**	65.4	30.4**	63.0
**e**_28_	40.2*	84.2	34.5**	66.9

Statistical significance of alignment was assessed by comparison to a null distribution of randomly generated pairs of vectors (see Methods); ^*^P < 0.05, ^**^P < 0.01, ^***^P < 0.001

The vector explaining the largest proportion of divergence in **G** (**e**_11_) was well aligned with the major axes of both morphological divergence (**d**_1_) and conserved genetic variance (**h**_1_; Table 4). Examination of first ten **e** vectors showed that divergence in **G** was more closely aligned with axes of conserved genetic variance (**h**_1_ and **h**_2_) than with axes of morphological divergence (**d**_1_ and **d**_2_; two‐tailed sign test, *P* = 0.04; Table S5).

**Figure 4:**
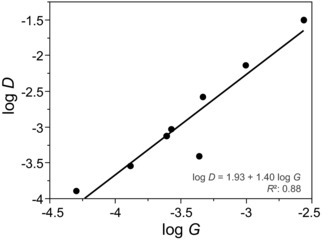
Relationship between log‐transformed genetic variance (*G*) and divergence (*D*) in a set of eight orthogonal trait combinations. Trait combinations are defined in a subspace common to the estimated ancestral G matrix (G_anc_) and divergence matrix (D) for seven *Anolis* species. Similar results were obtained when using other estimates of divergence (Fig. S2).

**Figure 5:**
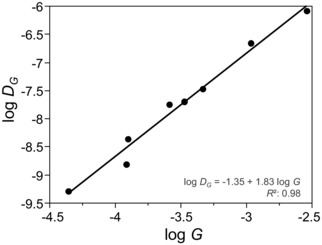
Relationship between log‐transformed genetic variance (*G*) and divergence in *G* (*D_G_*) in a set of eight orthogonal trait combinations defined by the estimated ancestral **G** matrix (**G**_anc_).

When we compared the estimated ancestral **G** matrix (**G**_anc_) to the divergence matrix **D** in a common subspace, we found a strong relationship between within‐species genetic variance (*G*) in a given direction and divergence in species means (*D*) in the same direction (Fig. 4; log‐log slope = 1.40 ± 0.208; *P* = 0.0005, *R* ^2^ = 0.88). In other words, trait combinations with more genetic variance showed greater divergence. The scaling relationship between *D* and *G* did not differ significantly from 1 (*P* = 0.102), a value predicted by various evolutionary models and observed in a recent study of fly wings (Houle et al. 2017). Similarly, divergence in **G** (*D_G_*) was also predicted by within‐species genetic variance (slope = 1.83 ± 0.105; *P* < 0.0001, *R* ^2^ = 0.98), with the trait combinations with the greatest genetic variance also showing the greatest divergence in variance across species (Fig. 5).

All results were similar when using alternative measures of species divergence (Table S6, Fig. S1).

## Discussion

Here, we present three main findings. First, we show that **G** has diverged substantially across the adaptive radiation of West Indian anoles, which is expected given that the seven species in our study are separated by over 40 million years. Second, we show that despite this divergence, all **G** matrices retain at least two similar axes of genetic variation and that the divergence of morphological trait means is biased toward the greatest of these (**h**_1_ or **g**_max_). This finding suggests that the evolutionary radiation of anole skeletal morphology proceeds along a genetic line of least resistance defined by **g**_max_. Third, we show that this pattern likely persisted because the evolution of **G** was proportional to both within‐species genetic variance and divergence in species means. In other words, evolution of **G** occurred in such a way as to preserve the relationship between axes of genetic variation and morphological divergence. Together, these findings suggest that groups of species may diverge along lines of genetic least resistance for millions of years and that this pattern is unlikely to be disrupted by concomitant changes in underlying genetic architecture.

The tight relationship between genetic variance within species and morphological divergence was surprising for two reasons. First, the relationship appears to have persisted despite divergence times of 20–40 million years. The relationship between divergence and **g**_max_ originally demonstrated by Schluter (1996) appeared to decay by around two million years, leading to the expectation that genetic architecture should be most important in the early stages of evolutionary radiation. Indeed, most studies demonstrating alignment between divergence and **g**_max_ have examined groups with divergence of two million years or less (Blows and Higgie 2003; Bégin and Roff 2004; McGuigan et al. 2005; Hansen and Houle 2008; Chenoweth et al. 2010; Bolstad et al. 2014; but see Houle et al. 2017). Second, the *Anolis* radiation has a well‐demonstrated adaptive basis, with replicate lineages repeatedly diversifying to fill common ecological niches on each of the Greater Antilles (Losos et al. 1998; Losos 2009; Mahler et al. 2013). Combined with similar results from a very different suite of morphological traits, drosophilid wing shape (Houle et al. 2017), our results suggest that alignment of genetic variance and species divergence may be more common and persist over longer timespans than previously expected.

Although the pattern demonstrated here is clear, the mechanisms underlying it are not. It is tempting to view this pattern as strong evidence that genetic constraints shape evolutionary change. However, the well‐established adaptive basis of morphological divergence in the *Anolis* radiation suggests that selection likely plays a role in generating this pattern. Below, we discuss potential mechanisms that may maintain a relationship between genetic variation and adaptive divergence across an ancient radiation.

### EVOLUTION ALONG GENETIC (OR SELECTIVE) LINES OF LEAST RESISTANCE

The persistence of the relationship between genetic variation and divergence despite both the age of the radiation and evidence for repeated adaptation suggests two plausible explanations, one emphasizing constraint and one emphasizing adaptation. First, *Anolis* species may diverge along genetic lines of least resistance simply because certain adaptive peaks happen to be more accessible genetically than others. In this view, there are many potential evolutionary optima available to anoles, but divergence tends to occur more often in certain directions with more available genetic variance. Much of the divergence among species in this study (as well as across all species of West Indian anoles, Beuttell and Losos 1999; Mahler et al. 2013) occurs by changes in overall limb length, and limb traits had consistently high genetic variances and positive genetic correlations across all species. This relationship suggests the possibility that anoles may be biased toward diverging in overall limb length—as opposed to other traits—by the availability of genetic variance in that direction. This view suggests that the repeated evolution of ecomorphs—which constitute ∼80% of anoles in the Greater Antilles (Losos 2009)—may have been favored by the genetic architecture of ancestral anoles.

An equally plausible scenario emphasizes selection as the ultimate factor underlying the alignment between **G** and **D**. In this view, the evolution of both species means and genetic variance are determined by “selective lines of least resistance” defined by the adaptive landscape (Arnold et al. 2001). Quantitative genetic theory predicts that **G** should eventually conform to the contours of the adaptive landscape (Cheverud 1982; Arnold et al. 2008). This process may be driven both by directional selection (i.e., movement of a population toward a new fitness peak on the adaptive landscape) and multivariate stabilizing selection (i.e., selection that stabilizes a population's occupancy of its current fitness peak) (Lande 1980; Cheverud 1982; Jones et al. 2003, 2004, 2007, 2012, 2014; Arnold et al. 2008).

For West Indian anoles, it is reasonable to expect that the adaptive landscape resembles a surface with multiple fitness peaks representing the ecomorphs that we see today (Mahler et al. 2013). Such an adaptive landscape could stabilize certain aspects of **G** (such as **g**_max_) and lead to the alignment between **G** and **D**. The repeated evolution of ecomorphs may resemble the repeated movement of fitness peaks along the same trait axis in response to interspecific competition (Schoener 1968; Williams 1972; Losos 1990b; Losos et al. 1994; Stuart and Losos 2013; Stuart et al. 2014). In simulation studies, this evolutionary scenario leads to an elongation of **G** in the direction of the moving optimum (Jones et al. 2004, 2012). As we discuss below, our results contain a signature of **G**‐matrix evolution consistent with this scenario, suggesting that selection is a more plausible source of the alignment of **G** and **D** than constraint.

### G‐MATRIX EVOLUTION AND MORPHOLOGICAL DIVERGENCE

Perhaps even more surprising than the correspondence between genetic variation and divergence is the fact that this alignment occurred despite evolutionary changes in the **G** matrix. Certain changes in **G**, such as dramatic alterations of its eigenstructure, would be expected to obscure the relationship between **G** and divergence. However, the observed changes in the **G** matrix across the seven *Anolis* species in this study occurred in a way that preserved the major axes of genetic variance. Genetic covariance tensor analysis showed that nearly half of the divergence in **G** could be accounted for by the first eigentensor (**E**_1_), which was highly correlated with the overall genetic variance. Further, 40% of divergence in **G** could be accounted for by change in genetic variance associated with a single combination of characters consisting primarily of limb‐length traits. This trait combination (**e**_11_) was highly aligned with both the first axis of divergence (**d**_1_) and the first axis of genetic variation (**h**_1_). These results suggest that a large portion of change in **G** can be interpreted as growing and shrinking of the **G** matrix along conserved axes of variation—including **g**_max_—as species means diverge along a genetic line of least resistance. Changes in **G**‐matrix shape and orientation also occurred, but did not obscure the relationship between divergence and **g**_max_. A similar pattern has recently been detected for **G**‐matrix evolution in a much younger (<1 million years) radiation of ecotypes within a plant species (*Senecio pinnatifolius*, Walter et al. 2018). Taken together, these results suggest that the alignment of phenotypic divergence and **G**‐matrix evolution may be a general phenomenon.

The alignment of divergence in **G** with both within‐population genetic variance and divergence of species means is likely to be a product of some combination of genetic constraint, drift, and selection. Although we cannot definitively distinguish among them, our results hint that each of the three mechanisms is likely to be at play.

#### Constraint

The relevant genetic constraint on the evolution of **G** is the mutational (co)variance matrix **M**, which describes the per‐generation input of new genetic variation in a population. The observed changes in **G** across species may reflect a deeper constraint on **G** imposed by the rate and phenotypic effect of mutations. Certain patterns within **M**, such as the correlation of mutational effects, may have a large effect on **G** even when opposed by selection (Jones et al. 2003). For example, if new mutations tend to have consistent pleiotropic effects, a genetic correlation between traits can be maintained even when selection does not favor a relationship between the traits.

Some of our observations, such as relative stability of orientation and more divergence of trait combinations with greater genetic variance, are consistent with a **G** matrix constrained by mutation. In drosophild flies, Houle et al. (2017) was able to estimate **M** in addition to **G** and **D**, demonstrating that both divergence and genetic variation could be predicted by mutation and suggesting a role for deep constraints in phenotypic evolution. We were unable to estimate **M** in anoles, but the combination of traits represented within **e**_11_ suggests that the generation of pleiotropic mutations may indeed play a role in how **G** diverges. This axis almost exclusively represents overall limb length, suggesting allelic variation in loci that pleiotropically affect the length of all limb bones (Leamy et al. 2002; Rabinowitz and Vokes 2012). The tendency for **G** to evolve in this direction could thus be biased by the tendency for mutations affecting limb length to be pleiotropic (Pavličev and Cheverud 2015).

#### Drift

Genetic drift is predicted to primarily influence **G**‐matrix size, with smaller populations retaining less genetic variance (Jones et al. 2003). **G** should thus change the most along **g**_max_ under drift alone. Consistent with this prediction, we found that **G** diverged primarily in size, and **G** matrices from the two larger islands (Puerto Rico and Jamaica), which likely harbor larger populations, were larger than those estimated for species collected from the small Bahamian island of South Bimini (the two species of Cuban origin). Drift should also cause population means to diverge in directions with more genetic variance, resulting in divergence along **g**_max_ and thus alignment between **d**_1_ and the direction of most change in **G**. Although this scenario is theoretically plausible, the well‐established role of selection in the evolution of *Anolis* ecomorphs (Losos 2009) suggests that neutral processes are highly unlikely to be the only factor explaining such alignment.

#### Selection

As we argued above, selection leading to the repeated evolution of ecomorphs is likely to influence **G**‐matrix evolution as well, which may lead to the observed triple‐alignment among genetic variance, morphological divergence, and divergence of **G**. There are at least three possible selective mechanisms at play. First, directional selection can cause the **G** matrix to grow in size when the evolutionary optimum moves along **g**_max_ (Jones et al. 2004, 2012). Such a process should not only stabilize the orientation of **g**_max_ but also cause changes in the magnitude of genetic variance explained by **g**_max_. The similarity of **h**_1_ to each species‐specific **g**_max_ and the alignment of **e**_11_ with both **h**_1_ and **d**_1_ are all consistent with this scenario. Second, multivariate nonlinear selection may further contribute to the stability of **G** by conforming its orientation to the adaptive landscape (Cheverud 1982; Jones et al. 2003). Such alignment could arise from similar curvature of the adaptive landscape surrounding the fitness peaks occupied by different species, which would be expected if selection favors similar patterns of phenotypic integration across microhabitats. A third plausible way that selection may contribute to evolution of **G** is by alteration of mutational constraints. Although our results cannot address this possibility, both theory and data suggest that the **M** matrix can evolve in response to selection, further stabilizing the alignment of **G** with the adaptive landscape (Jones et al. 2007, 2014; Houle et al. 2017).

## Conclusion

The repeated adaptive radiation of West Indian anoles illustrates that evolution may follow predictable pathways in response to similar ecological selection pressures (Losos et al. 1998; Mahler et al. 2013). Here, we have demonstrated that alignment between divergence and genetic variation—a pattern predicted to be generated by genetic constraints on evolution—persists in anoles despite over 40 million years of repeated adaptation to different ecological niches. This alignment echoes results from a recent study of a vastly different group of traits in flies (Houle et al. 2017), suggesting the pattern of radiation along genetic lines of least resistance may be common in nature, even when considering evolution over tens of millions of years. Contrary to expectations, the relationship between divergence and **G** persisted despite substantial evolution of **G** itself, because evolutionary changes in genetic architecture occurred in directions that did not disrupt the genetic line of least resistance.

Although we cannot definitively distinguish between genetic constraint, drift, and selection as the cause of this pattern, the alternatives lead to equally compelling conclusions about the evolutionary process. For example, our results could indicate that extensive adaptation is possible even in the face of genetic constraints that persist for tens of millions of years. Alternatively, the same patterns may suggest that genetic constraints themselves may be altered by selection, aligning genetic variation with the adaptive landscape and promoting evolutionary radiation. Further research is needed to determine whether the patterns demonstrated here are general and to dissect the mechanisms responsible for their persistence.

Associate Editor: A. Charmantier

## Supporting information


**Table S1**. Additive genetic (co)variance matrices (**G**) for seven *Anolis* species.
**Table S2**. Size‐corrected species means and divergence matrix.
**Table S3**. Divergence matrix for 15 *Anolis* species using traits measured in wild‐caught adult males.
**Table S4**. Krzanowski's common subspace analysis.
**Table S5**. Genetic covariance tensor analysis.
**Table S6**. Tests from Table 4 using alternative estimates of species divergence.
**Figure S1**. Species means of wild‐caught adult males along the first two divergence axes.
**Figure S2**. Relationship between log‐transformed genetic variance *(G)* and four different estimates of divergence in a set of eight orthogonal trait combinations.
