# Supplementary material for: Adaptive radiation along a deeply conserved genetic line of least resistance in Anolis lizards
Source: Evol Lett. 2018 Jul 17;2(4):310–22. doi: 10.1002/evl3.72 (PMC6121822; doi:10.1002/evl3.72)
Supplement: Supplementary file 1 — Table S1. Additive genetic (co)variance matrices (G) for seven Anolis species. Table S2. Size‐corrected species means and divergence matrix. Table S3. Divergence matrix for 15 Anolis species using traits measured in wild‐caught adult males. Table S4. Krzanowski's common subspace analysis. Table S5. Genetic covariance tensor analysis. Table S6. Tests from Table 4 using alternative estimates of species divergence. Figure S1. Species means of wild‐caught adult males along the first two divergence axes. Figure S2. Relationship between log‐transformed genetic variance (G) and four different estimates of divergence in a set of eight orthogonal trait combinations. [file EVL3-2-310-s001.docx]

**Supporting Information**

*Additional Methods*

**COLLECTION**

Adult lizards were captured from the wild under collecting permits from the proper government agencies and returned to a laboratory colony at the University of Virginia for breeding. We collected representatives of three different ecomorphs (trunk-crown, trunk-ground, and grass-bush) from lineages that originated independently on three different islands (Cuba, Puerto Rico, and Jamaica). Species from Cuban lineages were collected from South Bimini, The Bahamas (which is part of their native range), due to travel and permit restrictions. Lizards were collected on the following dates: *A. cristatellus*, November-December 2005, April 2006, and January-February 2008; *A. evermanni*, April 2006 and January-February 2008; *A. pulchellus*, January-February 2008; *A. sagrei* and *A. smaragdinus*, September 2006 and February 2009; *A. grahami* and *A. lineatopus*, April 2009. Import of animals from The Bahamas and Jamaica was approved by the United States Fish and Wildlife Service.

**HUSBANDRY AND BREEDING**

In the laboratory, adults were housed in individual cages (except when paired for breeding) in controlled environmental conditions with 12L:12D (for Puerto Rican and Jamaican adults) or 13L:11D (for Bahamian adults), a temperature of 28°C during the day and 25°C at night, and 65% relative humidity. All cages were lit with adjacent UVB bulbs, were lined with a carpet substrate, and contained a perch and mesh hammock for basking. Adults were fed crickets dusted with vitamins and minerals regularly and watered twice daily. All procedures were approved by the University of Virginia Animal Care and Use Committee.

We employed a predominantly paternal half-sib breeding design, with males typically mated to two different females. Pairs of the same individuals were reformed as needed to increase family size. In some cases (e.g., due to death of a mate or limited availability of females), females were remated to different males, leading to some maternal half-sibs in our dataset. Pairing began in December 2006 and ended in October 2010, with breeding of different species overlapping as lizards were collected from the field. For breeding, males were placed in a cage with a single female for ~4-6 weeks, after which time females were removed and placed in smaller cages. We ensured that females were not laying viable eggs from stored sperm before pairing. Females were provided with a small container of potting soil for depositing eggs. We checked for eggs weekly, and viable eggs were incubated in individual cups with a 1:1 mixture of water and vermiculite at 28°C and 80% humidity until hatching.

Hatchlings were placed in individual cages (Lee’s Kritter Keeper, 30 cm × 20 cm × 20 cm) in environmental conditions identical to those described above (with a photoperiod of 12L:12D). Juveniles were fed pinhead crickets (periodically dusted with vitamins and minerals) daily and watered twice daily. We reared juveniles until 6 months of age, when they were sacrificed and preserved in ethanol. All specimens are deposited at the Museum of Comparative Zoology at Harvard University.

**PHENOTYPING**

At 0, 1, 3, and 6 months, we obtained live X-ray images of each juvenile using a Faxitron 43805N radiography system. Juveniles were chilled for 10 min at 5°C within small zip-top plastic bags, which were subsequently secured to a film cartridge (Kodak Biomax XAR) for imaging. Images were digitized for analysis using ImageJ (NIH). For each image, we obtained nine measurements (in mm): snout-vent length (SVL), measured as a segmented line from the tip of the snout to the sacro-caudal junction; jaw length (JL), head width (HW), pectoral width (PECT), pelvic width (PELV), humerus length (HUM), ulna length (UL), femur length (FEM), and tibia length (TIB). Measurements were taken from the right side of the body when possible. In all, we measured 9,369 individual X-ray images.

**QUANTITATIVE GENETIC ANALYSES**

For each species, we estimated **G** using a multivariate repeated-measures animal model in ASReml (Gilmour et al. 2009) (Table S1). All morphological traits were natural-log transformed before analysis. Each model included all traits (except SVL) as response variables, natural-log SVL as a covariate (to control for size and growth), a pedigree-linked individual effect (to estimate **G**) and a non-pedigree-linked individual effect (to estimate permanent environment effects). We used approximate standard errors calculated by ASReml as guides to statistical robustness of our estimates. Permanent environment (co)variances were generally an order of magnitude (or more) smaller than genetic (co)variances, and are not presented here.

**ALTERNATIVE ESTIMATES OF SPECIES DIVERGENCE**

We also calculated two estimates of species divergence that accounted for phylogeny: the divergence matrix of phylogenetically independent contrasts (**D**_IC_, Revell et al. 2007) and a matrix of evolutionary rates (**R**) assuming a Brownian motion model of evolution. For these calculations, we used a seven-species tree pruned from a time-calibrated phylogeny of squamates (Zheng and Wiens 2016) and the R packages APE (Paradis et al. 2004) and mvMORPH (Clavel et al. 2015), respectively. These matrices were both highly similar to **D** (Table S2).

Although using **D** as an estimate of divergence is advantageous because all species means were measured in a common environment, it is potentially misleading because the number of species included is limited and because the use of juvenile traits may underestimate among-species variance. As a complement, we also calculated a divergence matrix using phenotypic data from 15 species of wild-caught adult males. This dataset included an independent sample of the seven species from which we measured **G** as well as eight additional species: *A. krugi* (grass-bush), *A. gundlachi* (trunk-ground), Puerto Rico; *A. garmani* (crown giant), Jamaica, *A. distichus* (trunk), *A. angusticeps* (twig), South Bimini; *A. coelestinus* (trunk-crown), *A. cybotes* (trunk-ground), *A. olssoni* (grass-bush), Hispaniola. All males were sacrificed after capture and radiographs of preserved specimens were taken and measured as above. We fit linear regressions of natural-log transformed trait values on natural-log SVL to obtain estimates of intercepts and slopes to calculate size-corrected species means using the grand mean SVL. These size-corrected means were used to calculate a divergence matrix (**D**_15_, Table S3).

**LITERATURE CITED**

Clavel, J., G. Escarguel, and G. Merceron. 2015. mvMORPH: an R package for fitting multivariate evolutionary models to morphometric data. Methods Ecol Evol 6:1311-1319.

Gilmour, A. R., B. J. Gogel, B. R. Cullis, and R. Thompson. 2009. ASReml User Guide Release 3.0. VSN International Ltd, Hemel Hempstead, UK.

Hine, E., S. F. Chenoweth, H. D. Rundle, and M. W. Blows. 2009. Characterizing the evolution of genetic variance using genetic covariance tensors. Philos. Trans. R. Soc. Lond. B 364:1567-1578.

Paradis, E., J. Claude, and K. Strimmer. 2004. APE: Analyses of Phylogenetics and Evolution in R language. Bioinformatics 20:289-290.

Revell, L. J., L. J. Harmon, R. B. Langerhans, and J. J. Kolbe. 2007. A phylogenetic approach to determining the importance of constraint on phenotypic evolution in the neotropical lizard Anolis cristatellus. Evol. Ecol. Res. 9:261-282.

Zheng, Y. C., and J. J. Wiens. 2016. Combining phylogenomic and supermatrix approaches, and a time-calibrated phylogeny for squamate reptiles (lizards and snakes) based on 52 genes and 4162 species. Mol. Phylogenet. Evol. 94:537-547.

*Additional Tables and Figures*

**Table S1**. Additive genetic (co)variance matrices (**G**) for seven *Anolis* species. **G** is multiplied by 10^3^ for clarity. For each **G**, genetic variances are on the diagonal and genetic covariances are off the diagonal. Standard errors, multiplied by 10^3^ for clarity, and the results of eigenanalysis are shown below each **G** matrix. Parameters that exceeded their standard errors by a factor of two are shown in bold. In this and the tables that follow, traits are abbreviated as follows: JL = jaw length, HW = head width, PECT = pectoral width, PELV = pelvic width, HUM = humerus, UL = ulna, FEM = femur, and TIB = tibia. All traits were natural-log transformed and size-corrected for analysis. An ancestral **G** for the common ancestor of all 7 species was estimated using maximum likelihood and a Brownian motion model of evolution.

*Anolis cristatellus* (Trunk-ground, Puerto Rico)

**G** (× 10^-3^)

|  | JL | HW | PECT | PELV | HUM | UL | FEM | TIB |
| --- | --- | --- | --- | --- | --- | --- | --- | --- |
| JL | **0.329** |  |  |  |  |  |  |  |
| HW | **0.094** | **0.449** |  |  |  |  |  |  |
| PECT | -0.083 | **0.349** | **1.426** |  |  |  |  |  |
| PELV | -0.089 | **0.240** | **0.487** | **0.546** |  |  |  |  |
| HUM | **0.293** | 0.071 | **-0.371** | **-0.168** | **1.441** |  |  |  |
| UL | 0.079 | 0.075 | -0.098 | 0.017 | **1.008** | **1.087** |  |  |
| FEM | **0.208** | 0.030 | -0.053 | -0.051 | **0.904** | **0.731** | **0.809** |  |
| TIB | **0.268** | 0.009 | **-0.172** | -0.081 | **0.945** | **0.780** | **0.783** | **0.949** |

SE (× 10^-3^)

|  | JL | HW | PECT | PELV | HUM | UL | FEM | TIB |
| --- | --- | --- | --- | --- | --- | --- | --- | --- |
| JL | 0.052 |  |  |  |  |  |  |  |
| HW | 0.029 | 0.048 |  |  |  |  |  |  |
| PECT | 0.053 | 0.066 | 0.170 |  |  |  |  |  |
| PELV | 0.056 | 0.049 | 0.093 | 0.113 |  |  |  |  |
| HUM | 0.057 | 0.067 | 0.128 | 0.090 | 0.200 |  |  |  |
| UL | 0.053 | 0.063 | 0.119 | 0.087 | 0.144 | 0.175 |  |  |
| FEM | 0.035 | 0.042 | 0.077 | 0.058 | 0.093 | 0.085 | 0.071 |  |
| TIB | 0.037 | 0.043 | 0.081 | 0.060 | 0.096 | 0.089 | 0.067 | 0.076 |

Eigenvalues and eigenvectors

|  | **g**_max_ | **g**_2_ | **g**_3_ | **g**_4_ | **g**_5_ | **g**_6_ | **g**_7_ | **g**_8_ |
| --- | --- | --- | --- | --- | --- | --- | --- | --- |
| *λ* (× 10^-3^) | 3.83 | 1.74 | 0.460 | 0.424 | 0.264 | 0.158 | 0.096 | 0.067 |
| % var | 54.36 | 24.76 | 6.54 | 6.02 | 3.76 | 2.24 | 1.37 | 0.96 |
| JL | 0.126 | -0.009 | 0.681 | 0.118 | 0.152 | 0.129 | 0.547 | 0.427 |
| HW | 0.008 | 0.311 | 0.281 | 0.677 | -0.234 | 0.349 | -0.418 | -0.123 |
| PECT | -0.170 | 0.835 | 0.129 | -0.425 | -0.232 | -0.052 | 0.118 | -0.062 |
| PELV | -0.074 | 0.394 | -0.314 | 0.461 | 0.601 | -0.365 | 0.172 | 0.060 |
| HUM | 0.586 | 0.003 | 0.089 | 0.183 | -0.404 | -0.606 | 0.133 | -0.236 |
| UL | 0.468 | 0.164 | -0.547 | 0.059 | -0.211 | 0.496 | 0.309 | 0.285 |
| FEM | 0.421 | 0.134 | 0.095 | -0.235 | 0.224 | -0.159 | -0.608 | 0.536 |
| TIB | 0.458 | 0.076 | 0.162 | -0.207 | 0.499 | 0.296 | 0.002 | -0.610 |

*Anolis evermanni* (Trunk-crown, Puerto Rico)

**G** (× 10^-3^)

|  | JL | HW | PECT | PELV | HUM | UL | FEM | TIB |
| --- | --- | --- | --- | --- | --- | --- | --- | --- |
| JL | **0.251** |  |  |  |  |  |  |  |
| HW | -0.013 | **0.314** |  |  |  |  |  |  |
| PECT | 0.016 | **0.411** | **1.062** |  |  |  |  |  |
| PELV | -0.017 | **0.183** | **0.435** | **0.623** |  |  |  |  |
| HUM | **0.143** | 0.022 | 0.051 | 0.139 | **0.866** |  |  |  |
| UL | **0.141** | -0.026 | 0.097 | 0.145 | **0.884** | **0.971** |  |  |
| FEM | 0.070 | 0.040 | **0.228** | **0.279** | **0.850** | **0.922** | **1.224** |  |
| TIB | **0.121** | 0.047 | 0.178 | **0.258** | **0.971** | **1.064** | **1.427** | **1.751** |

SE (× 10^-3^)

|  | JL | HW | PECT | PELV | HUM | UL | FEM | TIB |
| --- | --- | --- | --- | --- | --- | --- | --- | --- |
| JL | 0.029 |  |  |  |  |  |  |  |
| HW | 0.027 | 0.049 |  |  |  |  |  |  |
| PECT | 0.048 | 0.069 | 0.163 |  |  |  |  |  |
| PELV | 0.040 | 0.054 | 0.099 | 0.146 |  |  |  |  |
| HUM | 0.049 | 0.061 | 0.110 | 0.091 | 0.160 |  |  |  |
| UL | 0.049 | 0.062 | 0.113 | 0.093 | 0.135 | 0.156 |  |  |
| FEM | 0.041 | 0.054 | 0.098 | 0.083 | 0.108 | 0.110 | 0.117 |  |
| TIB | 0.048 | 0.064 | 0.114 | 0.097 | 0.124 | 0.128 | 0.125 | 0.174 |

Eigenvalues and eigenvectors

|  | **g**_max_ | **g**_2_ | **g**_3_ | **g**_4_ | **g**_5_ | **g**_6_ | **g**_7_ | **g**_8_ |
| --- | --- | --- | --- | --- | --- | --- | --- | --- |
| *λ* (× 10^-3^) | 4.46 | 1.45 | 0.42 | 0.34 | 0.21 | 0.15 | 0.029 | 0.006 |
| % var | 63.13 | 20.49 | 5.89 | 4.95 | 2.93 | 2.12 | 0.41 | 0.08 |
| JL | 0.054 | 0.036 | 0.414 | 0.006 | -0.889 | 0.099 | 0.095 | 0.131 |
| HW | 0.027 | -0.360 | 0.088 | -0.167 | 0.143 | 0.836 | -0.090 | 0.311 |
| PECT | 0.104 | -0.801 | 0.255 | -0.333 | 0.031 | -0.376 | 0.026 | -0.175 |
| PELV | 0.121 | -0.440 | -0.319 | 0.815 | -0.158 | 0.033 | -0.065 | -0.020 |
| HUM | 0.399 | 0.123 | 0.460 | 0.242 | 0.233 | 0.243 | 0.413 | -0.513 |
| UL | 0.430 | 0.120 | 0.447 | 0.190 | 0.226 | -0.231 | -0.540 | 0.402 |
| FEM | 0.513 | 0.000 | -0.277 | -0.115 | 0.027 | -0.139 | 0.601 | 0.528 |
| TIB | 0.603 | 0.071 | -0.408 | -0.298 | -0.239 | 0.135 | -0.394 | -0.389 |

*Anolis grahami* (Trunk-crown, Jamaica)

**G** (× 10^-3^)

|  | JL | HW | PECT | PELV | HUM | UL | FEM | TIB |
| --- | --- | --- | --- | --- | --- | --- | --- | --- |
| JL | **0.246** |  |  |  |  |  |  |  |
| HW | **0.218** | **0.296** |  |  |  |  |  |  |
| PECT | 0.127 | 0.122 | **0.351** |  |  |  |  |  |
| PELV | **0.193** | 0.126 | 0.173 | **0.704** |  |  |  |  |
| HUM | 0.152 | 0.067 | 0.167 | 0.264 | **1.003** |  |  |  |
| UL | 0.129 | 0.131 | 0.122 | 0.130 | **0.856** | **1.171** |  |  |
| FEM | **0.126** | -0.005 | 0.081 | 0.186 | **0.625** | **0.559** | **0.510** |  |
| TIB | **0.146** | 0.034 | 0.171 | **0.269** | **0.732** | **0.724** | **0.549** | **0.648** |

SE (× 10^-3^)

|  | JL | HW | PECT | PELV | HUM | UL | FEM | TIB |
| --- | --- | --- | --- | --- | --- | --- | --- | --- |
| JL | 0.049 |  |  |  |  |  |  |  |
| HW | 0.051 | 0.082 |  |  |  |  |  |  |
| PECT | 0.064 | 0.081 | 0.151 |  |  |  |  |  |
| PELV | 0.068 | 0.084 | 0.113 | 0.169 |  |  |  |  |
| HUM | 0.140 | 0.186 | 0.238 | 0.176 | 0.465 |  |  |  |
| UL | 0.103 | 0.133 | 0.177 | 0.186 | 0.323 | 0.405 |  |  |
| FEM | 0.052 | 0.099 | 0.139 | 0.111 | 0.230 | 0.191 | 0.170 |  |
| TIB | 0.056 | 0.102 | 0.145 | 0.104 | 0.230 | 0.181 | 0.163 | 0.179 |

Eigenvalues and eigenvectors

|  | **g**_max_ | **g**_2_ | **g**_3_ | **g**_4_ | **g**_5_ | **g**_6_ | **g**_7_ | **g**_8_ |
| --- | --- | --- | --- | --- | --- | --- | --- | --- |
| *λ* (× 10^-3^) | 3.08 | 0.82 | 0.44 | 0.26 | 0.20 | 0.12 | 0.011 | 0.003 |
| % var | 62.55 | 16.58 | 8.87 | 5.27 | 4.09 | 2.35 | 0.21 | 0.06 |
| JL | 0.117 | 0.341 | 0.330 | 0.002 | 0.484 | 0.280 | -0.669 | 0.045 |
| HW | 0.066 | 0.314 | 0.628 | -0.165 | 0.268 | -0.184 | 0.598 | -0.135 |
| PECT | 0.117 | 0.329 | 0.260 | 0.751 | -0.453 | 0.045 | 0.002 | 0.170 |
| PELV | 0.191 | 0.754 | -0.405 | -0.393 | -0.258 | -0.061 | 0.009 | 0.083 |
| HUM | 0.540 | -0.082 | -0.155 | 0.210 | 0.270 | -0.738 | -0.138 | 0.028 |
| UL | 0.552 | -0.310 | 0.389 | -0.414 | -0.461 | 0.110 | -0.151 | 0.195 |
| FEM | 0.369 | -0.066 | -0.255 | 0.134 | 0.371 | 0.447 | 0.370 | 0.534 |
| TIB | 0.447 | -0.007 | -0.157 | 0.142 | 0.000 | 0.354 | 0.129 | -0.787 |

*Anolis lineatopus* (Trunk-ground, Jamaica)

**G** (× 10^-3^)

|  | JL | HW | PECT | PELV | HUM | UL | FEM | TIB |
| --- | --- | --- | --- | --- | --- | --- | --- | --- |
| JL | **0.442** |  |  |  |  |  |  |  |
| HW | **0.286** | **0.263** |  |  |  |  |  |  |
| PECT | **0.385** | **0.290** | **0.750** |  |  |  |  |  |
| PELV | 0.193 | 0.132 | 0.343 | **0.916** |  |  |  |  |
| HUM | **0.321** | **0.240** | 0.223 | -0.016 | **0.968** |  |  |  |
| UL | **0.412** | **0.297** | 0.308 | -0.200 | **0.942** | **1.047** |  |  |
| FEM | **0.423** | 0.180 | **0.547** | 0.228 | **0.530** | **0.540** | **1.013** |  |
| TIB | **0.368** | 0.145 | **0.528** | 0.182 | **0.464** | **0.495** | **0.864** | **0.825** |

SE (× 10^-3^)

|  | JL | HW | PECT | PELV | HUM | UL | FEM | TIB |
| --- | --- | --- | --- | --- | --- | --- | --- | --- |
| JL | 0.103 |  |  |  |  |  |  |  |
| HW | 0.059 | 0.090 |  |  |  |  |  |  |
| PECT | 0.101 | 0.100 | 0.309 |  |  |  |  |  |
| PELV | 0.158 | 0.139 | 0.254 | 0.352 |  |  |  |  |
| HUM | 0.127 | 0.103 | 0.213 | 0.200 | 0.291 |  |  |  |
| UL | 0.106 | 0.104 | 0.186 | 0.174 | 0.240 | 0.270 |  |  |
| FEM | 0.168 | 0.130 | 0.233 | 0.256 | 0.255 | 0.248 | 0.346 |  |
| TIB | 0.147 | 0.117 | 0.212 | 0.227 | 0.226 | 0.222 | 0.295 | 0.272 |

Eigenvalues and eigenvectors

|  | **g**_max_ | **g**_2_ | **g**_3_ | **g**_4_ | **g**_5_ | **g**_6_ | **g**_7_ | **g**_8_ |
| --- | --- | --- | --- | --- | --- | --- | --- | --- |
| *λ* (× 10^-3^) | 3.52 | 1.39 | 0.64 | 0.43 | 0.17 | 0.051 | 0.020 | 0.005 |
| % var | 56.63 | 22.37 | 10.23 | 6.91 | 2.65 | 0.82 | 0.33 | 0.08 |
| JL | 0.282 | 0.095 | 0.193 | 0.324 | -0.679 | 0.291 | 0.229 | -0.425 |
| HW | 0.174 | 0.035 | 0.338 | 0.370 | -0.217 | -0.358 | -0.665 | 0.324 |
| PECT | 0.327 | 0.352 | 0.032 | 0.568 | 0.608 | -0.081 | 0.176 | -0.196 |
| PELV | 0.107 | 0.661 | 0.520 | -0.467 | 0.022 | 0.104 | 0.100 | 0.193 |
| HUM | 0.418 | -0.388 | 0.317 | -0.391 | 0.229 | -0.157 | -0.221 | -0.542 |
| UL | 0.445 | -0.469 | 0.225 | 0.058 | 0.062 | 0.215 | 0.378 | 0.575 |
| FEM | 0.470 | 0.182 | -0.468 | -0.217 | -0.251 | -0.598 | 0.226 | 0.090 |
| TIB | 0.423 | 0.160 | -0.454 | -0.122 | 0.054 | 0.585 | -0.471 | 0.082 |

*Anolis pulchellus* (Grass-bush, Puerto Rico)

**G** (× 10^-3^)

|  | JL | HW | PECT | PELV | HUM | UL | FEM | TIB |
| --- | --- | --- | --- | --- | --- | --- | --- | --- |
| JL | 0.084 |  |  |  |  |  |  |  |
| HW | **0.080** | **0.157** |  |  |  |  |  |  |
| PECT | **0.222** | **0.215** | **0.895** |  |  |  |  |  |
| PELV | 0.070 | 0.065 | 0.300 | **0.766** |  |  |  |  |
| HUM | **0.148** | 0.040 | 0.153 | -0.036 | **0.745** |  |  |  |
| UL | **0.180** | 0.080 | 0.214 | -0.152 | **0.773** | **0.982** |  |  |
| FEM | 0.092 | 0.040 | 0.156 | 0.126 | **0.475** | **0.524** | **0.701** |  |
| TIB | 0.089 | 0.030 | 0.136 | 0.134 | **0.403** | **0.524** | **0.648** | **0.723** |

SE (× 10^-3^)

|  | JL | HW | PECT | PELV | HUM | UL | FEM | TIB |
| --- | --- | --- | --- | --- | --- | --- | --- | --- |
| JL | 0.050 |  |  |  |  |  |  |  |
| HW | 0.040 | 0.059 |  |  |  |  |  |  |
| PECT | 0.075 | 0.070 | 0.268 |  |  |  |  |  |
| PELV | 0.066 | 0.061 | 0.192 | 0.244 |  |  |  |  |
| HUM | 0.072 | 0.068 | 0.163 | 0.127 | 0.204 |  |  |  |
| UL | 0.082 | 0.076 | 0.155 | 0.145 | 0.197 | 0.254 |  |  |
| FEM | 0.054 | 0.053 | 0.110 | 0.083 | 0.133 | 0.118 | 0.156 |  |
| TIB | 0.054 | 0.045 | 0.087 | 0.082 | 0.141 | 0.143 | 0.148 | 0.168 |

Eigenvalues and eigenvectors

|  | **g**_max_ | **g**_2_ | **g**_3_ | **g**_4_ | **g**_5_ | **g**_6_ | **g**_7_ | **g**_8_ |
| --- | --- | --- | --- | --- | --- | --- | --- | --- |
| *λ* (× 10^-3^) | 2.60 | 1.17 | 0.72 | 0.34 | 0.11 | 0.092 | 0.025 | 0.002 |
| % var | 51.42 | 23.21 | 14.17 | 6.69 | 2.18 | 1.82 | 0.49 | 0.04 |
| JL | 0.123 | 0.128 | 0.162 | 0.036 | 0.169 | 0.062 | 0.188 | -0.940 |
| HW | 0.064 | 0.167 | 0.176 | -0.094 | 0.692 | 0.603 | 0.109 | 0.235 |
| PECT | 0.222 | 0.667 | 0.528 | -0.328 | -0.288 | -0.130 | 0.000 | 0.141 |
| PELV | 0.051 | 0.651 | -0.455 | 0.574 | 0.119 | -0.096 | -0.113 | 0.034 |
| HUM | 0.472 | -0.173 | 0.168 | 0.497 | -0.353 | 0.292 | 0.490 | 0.139 |
| UL | 0.556 | -0.239 | 0.294 | 0.223 | 0.350 | -0.381 | -0.478 | 0.046 |
| FEM | 0.451 | 0.006 | -0.384 | -0.292 | -0.306 | 0.493 | -0.468 | -0.120 |
| TIB | 0.440 | 0.008 | -0.442 | -0.413 | 0.234 | -0.365 | 0.503 | 0.070 |

*Anolis sagrei* (Trunk-ground, Cuba/Bahamas)

**G** (× 10^-3^)

|  | JL | HW | PECT | PELV | HUM | UL | FEM | TIB |
| --- | --- | --- | --- | --- | --- | --- | --- | --- |
| JL | **0.292** |  |  |  |  |  |  |  |
| HW | **0.112** | **0.302** |  |  |  |  |  |  |
| PECT | -0.002 | **0.166** | **0.286** |  |  |  |  |  |
| PELV | -0.077 | -0.018 | 0.035 | **0.463** |  |  |  |  |
| HUM | **0.156** | -0.073 | 0.027 | -0.109 | **0.603** |  |  |  |
| UL | 0.027 | **-0.142** | -0.047 | 0.082 | **0.462** | **0.646** |  |  |
| FEM | **0.192** | -0.033 | -0.027 | -0.011 | **0.409** | **0.315** | **0.368** |  |
| TIB | **0.177** | **-0.111** | -0.036 | -0.003 | **0.527** | **0.507** | **0.434** | **0.606** |

SE (× 10^-3^)

|  | JL | HW | PECT | PELV | HUM | UL | FEM | TIB |
| --- | --- | --- | --- | --- | --- | --- | --- | --- |
| JL | 0.023 |  |  |  |  |  |  |  |
| HW | 0.021 | 0.033 |  |  |  |  |  |  |
| PECT | 0.024 | 0.030 | 0.048 |  |  |  |  |  |
| PELV | 0.050 | 0.055 | 0.043 | 0.073 |  |  |  |  |
| HUM | 0.035 | 0.050 | 0.050 | 0.068 | 0.092 |  |  |  |
| UL | 0.037 | 0.044 | 0.053 | 0.062 | 0.080 | 0.116 |  |  |
| FEM | 0.024 | 0.037 | 0.042 | 0.041 | 0.051 | 0.051 | 0.054 |  |
| TIB | 0.026 | 0.044 | 0.051 | 0.042 | 0.058 | 0.061 | 0.056 | 0.073 |

Eigenvalues and eigenvectors

|  | **g**_max_ | **g**_2_ | **g**_3_ | **g**_4_ | **g**_5_ | **g**_6_ | **g**_7_ | **g**_8_ |
| --- | --- | --- | --- | --- | --- | --- | --- | --- |
| *λ* (× 10^-3^) | 1.97 | 0.60 | 0.47 | 0.29 | 0.13 | 0.054 | 0.042 | 0.010 |
| % var | 55.26 | 16.91 | 13.14 | 8.00 | 3.69 | 1.53 | 1.15 | 0.30 |
| JL | 0.153 | 0.452 | 0.143 | 0.569 | -0.125 | 0.340 | 0.550 | 0.005 |
| HW | -0.101 | 0.437 | 0.505 | -0.030 | -0.524 | -0.354 | -0.336 | -0.189 |
| PECT | -0.034 | 0.217 | 0.546 | -0.565 | 0.341 | 0.405 | 0.105 | 0.219 |
| PELV | -0.022 | -0.615 | 0.645 | 0.347 | 0.172 | -0.157 | 0.115 | -0.139 |
| HUM | 0.519 | 0.182 | -0.022 | -0.260 | 0.288 | -0.531 | 0.364 | -0.350 |
| UL | 0.496 | -0.357 | 0.048 | -0.281 | -0.647 | 0.132 | 0.220 | 0.249 |
| FEM | 0.397 | 0.137 | 0.082 | 0.273 | 0.214 | -0.271 | -0.303 | 0.715 |
| TIB | 0.540 | -0.005 | 0.033 | 0.119 | 0.128 | 0.444 | -0.537 | -0.448 |

*Anolis smaragdinus* (Trunk-crown, Cuba/Bahamas)

**G** (× 10^‑3^)

|  | JL | HW | PECT | PELV | HUM | UL | FEM | TIB |
| --- | --- | --- | --- | --- | --- | --- | --- | --- |
| JL | **0.348** |  |  |  |  |  |  |  |
| HW | 0.054 | 0.161 |  |  |  |  |  |  |
| PECT | 0.251 | 0.065 | **0.830** |  |  |  |  |  |
| PELV | 0.102 | -0.002 | **0.442** | **0.560** |  |  |  |  |
| HUM | **0.268** | 0.151 | 0.130 | 0.122 | 0.395 |  |  |  |
| UL | **0.238** | 0.094 | 0.249 | 0.300 | 0.331 | 0.452 |  |  |
| FEM | 0.205 | 0.061 | 0.136 | 0.115 | 0.285 | 0.287 | **0.344** |  |
| TIB | 0.296 | 0.008 | 0.221 | 0.278 | **0.351** | **0.423** | **0.420** | **0.609** |

SE (× 10^-3^)

|  | JL | HW | PECT | PELV | HUM | UL | FEM | TIB |
| --- | --- | --- | --- | --- | --- | --- | --- | --- |
| JL | 0.129 |  |  |  |  |  |  |  |
| HW | 0.091 | 0.139 |  |  |  |  |  |  |
| PECT | 0.171 | 0.174 | 0.413 |  |  |  |  |  |
| PELV | 0.072 | 0.068 | 0.144 | 0.142 |  |  |  |  |
| HUM | 0.130 | 0.093 | 0.163 | 0.122 | 0.280 |  |  |  |
| UL | 0.098 | 0.134 | 0.176 | 0.175 | 0.181 | 0.286 |  |  |
| FEM | 0.114 | 0.085 | 0.184 | 0.127 | 0.149 | 0.149 | 0.165 |  |
| TIB | 0.125 | 0.072 | 0.205 | 0.141 | 0.120 | 0.132 | 0.158 | 0.197 |

Eigenvalues and eigenvectors

|  | **g**_max_ | **g**_2_ | **g**_3_ | **g**_4_ | **g**_5_ | **g**_6_ | **g**_7_ | **g**_8_ |
| --- | --- | --- | --- | --- | --- | --- | --- | --- |
| *λ* (× 10^-3^) | 2.12 | 0.80 | 0.35 | 0.23 | 0.12 | 0.057 | 0.015 | 0.005 |
| % var | 57.30 | 21.55 | 9.50 | 6.27 | 3.28 | 1.55 | 0.41 | 0.15 |
| JL | 0.304 | 0.113 | 0.426 | 0.298 | 0.675 | 0.078 | 0.384 | 0.131 |
| HW | 0.080 | 0.069 | 0.381 | -0.578 | -0.284 | 0.129 | 0.524 | -0.332 |
| PECT | 0.411 | -0.703 | 0.404 | 0.206 | -0.277 | -0.117 | -0.199 | -0.067 |
| PELV | 0.350 | -0.434 | -0.564 | -0.317 | 0.233 | 0.403 | 0.202 | 0.148 |
| HUM | 0.338 | 0.324 | 0.264 | -0.350 | 0.106 | 0.377 | -0.647 | 0.034 |
| UL | 0.414 | 0.135 | -0.148 | -0.353 | 0.121 | -0.798 | -0.035 | 0.127 |
| FEM | 0.324 | 0.301 | -0.024 | 0.208 | -0.539 | 0.131 | 0.278 | 0.633 |
| TIB | 0.469 | 0.295 | -0.318 | 0.380 | -0.131 | 0.072 | -0.007 | -0.654 |

*Anolis sp.* (Ancestral **G**, estimated)

**G** (× 10^‑3^)

|  | JL | HW | PECT | PELV | HUM | UL | FEM | TIB |
| --- | --- | --- | --- | --- | --- | --- | --- | --- |
| JL | 0.285 |  |  |  |  |  |  |  |
| HW | 0.118 | 0.277 |  |  |  |  |  |  |
| PECT | 0.131 | 0.230 | 0.800 |  |  |  |  |  |
| PELV | 0.053 | 0.103 | 0.317 | 0.654 |  |  |  |  |
| HUM | 0.212 | 0.074 | 0.054 | 0.028 | 0.858 |  |  |  |
| UL | 0.172 | 0.073 | 0.121 | 0.047 | 0.749 | 0.906 |  |  |
| FEM | 0.188 | 0.045 | 0.152 | 0.124 | 0.581 | 0.553 | 0.708 |  |
| TIB | 0.210 | 0.023 | 0.147 | 0.148 | 0.626 | 0.644 | 0.731 | 0.872 |

Eigenvalues and eigenvectors

|  | **g**_max_ | **g**_2_ | **g**_3_ | **g**_4_ | **g**_5_ | **g**_6_ | **g**_7_ | **g**_8_ |
| --- | --- | --- | --- | --- | --- | --- | --- | --- |
| *λ* (× 10^-3^) | 2.91 | 1.09 | 0.47 | 0.34 | 0.26 | 0.13 | 0.12 | 0.04 |
| % var | 54.22 | 20.37 | 8.75 | 6.33 | 4.87 | 2.35 | 2.28 | 0.83 |
| JL | 0.157 | 0.100 | 0.193 | -0.120 | 0.703 | -0.498 | -0.397 | 0.120 |
| HW | 0.063 | 0.262 | 0.352 | 0.118 | 0.465 | 0.745 | 0.083 | -0.106 |
| PECT | 0.141 | 0.738 | 0.412 | -0.265 | -0.380 | -0.203 | 0.093 | -0.036 |
| PELV | 0.101 | 0.562 | -0.634 | 0.506 | 0.109 | -0.049 | -0.015 | 0.034 |
| HUM | 0.487 | -0.193 | 0.199 | 0.347 | 0.115 | -0.297 | 0.646 | -0.215 |
| UL | 0.496 | -0.144 | 0.257 | 0.439 | -0.342 | 0.122 | -0.546 | 0.207 |
| FEM | 0.451 | -0.018 | -0.239 | -0.403 | 0.051 | 0.184 | 0.249 | 0.691 |
| TIB | 0.504 | -0.037 | -0.323 | -0.412 | -0.012 | 0.130 | -0.221 | -0.636 |

**Table S2.** Size-corrected species means and divergence matrix. Species means were calculated from model parameters to correct for species differences in size. We used ln SVL = 3.55 to calculate size-corrected species means. Model parameters and back-transformed means (in mm) are presented below. The variance-covariance matrix uses natural-log transformed traits. Eigenanalysis of **D** and species coordinates along each eigenvector are shown. Species are listed using the first four letters of their specific name.

Parameters used to calculate species means

|  | Species | | | | | | |
| --- | --- | --- | --- | --- | --- | --- | --- |
|  | Intercepts | |  |  |  |  |  |
| Trait | CRIS | EVER | GRAH | LINE | PULC | SAGR | SMAR |
| JL | -0.9246 | -0.9602 | -0.9142 | -0.9237 | -0.9799 | -0.6587 | -1.017 |
| HW | -0.674 | -0.5792 | -0.6288 | -0.6186 | -0.4984 | -0.6633 | -0.454 |
| PECT | -1.283 | -1.934 | -2.108 | -1.619 | -1.609 | -1.724 | -1.975 |
| PELV | -2.272 | -2.471 | -2.408 | -2.298 | -2.234 | -2.295 | -2.417 |
| HUM | -2.711 | -2.467 | -2.373 | -2.494 | -2.567 | -2.686 | -2.217 |
| UL | -3.052 | -2.552 | -2.67 | -2.69 | -2.952 | -3.136 | -2.805 |
| FEM | -2.211 | -1.959 | -1.995 | -1.901 | -1.934 | -2.187 | -1.952 |
| TIB | -2.339 | -2.162 | -2.154 | -2.003 | -2.074 | -2.347 | -2.138 |
|  |  |  |  |  |  |  |  |
|  | Slopes |  |  |  |  |  |  |
| Trait | CRIS | EVER | GRAH | LINE | PULC | SAGR | SMAR |
| JL | 0.9462 | 0.9476 | 0.9436 | 0.9434 | 0.9547 | 0.8514 | 0.9682 |
| HW | 0.6897 | 0.6449 | 0.6665 | 0.6631 | 0.5773 | 0.665 | 0.5871 |
| PECT | 0.7996 | 0.9739 | 1.043 | 0.8795 | 0.8578 | 0.9279 | 0.9819 |
| PELV | 0.9982 | 1.047 | 1.033 | 0.9811 | 0.9368 | 0.9804 | 1.018 |
| HUM | 1.239 | 1.176 | 1.122 | 1.158 | 1.154 | 1.218 | 1.061 |
| UL | 1.275 | 1.142 | 1.141 | 1.163 | 1.192 | 1.274 | 1.139 |
| FEM | 1.219 | 1.122 | 1.126 | 1.127 | 1.11 | 1.185 | 1.08 |
| TIB | 1.225 | 1.158 | 1.145 | 1.128 | 1.118 | 1.202 | 1.081 |

Back-transformed species means (mm)

|  | Species | | | | | | |
| --- | --- | --- | --- | --- | --- | --- | --- |
| Trait | CRIS | EVER | GRAH | LINE | PULC | SAGR | SMAR |
| JL | 11.409 | 11.065 | 11.422 | 11.306 | 11.126 | 10.631 | 11.247 |
| HW | 5.900 | 5.530 | 5.682 | 5.671 | 4.716 | 5.460 | 5.105 |
| PECT | 4.736 | 4.587 | 4.926 | 4.496 | 4.205 | 4.807 | 4.530 |
| PELV | 3.565 | 3.476 | 3.522 | 3.270 | 2.979 | 3.272 | 3.310 |
| HUM | 5.400 | 5.517 | 5.003 | 5.037 | 4.617 | 5.144 | 4.709 |
| UL | 4.377 | 4.491 | 3.977 | 4.215 | 3.595 | 4.001 | 3.450 |
| FEM | 8.301 | 7.569 | 7.406 | 8.165 | 7.437 | 7.536 | 6.566 |
| TIB | 7.469 | 7.021 | 6.758 | 7.399 | 6.652 | 6.821 | 5.471 |

Variance-covariance matrix of species means (**D**, × 10^-3^)

|  | JL | HW | PECT | PELV | HUM | UL | FEM | TIB |
| --- | --- | --- | --- | --- | --- | --- | --- | --- |
| JL | 0.616 |  |  |  |  |  |  |  |
| HW | 0.498 | 5.81 |  |  |  |  |  |  |
| PECT | -0.004 | 3.06 | 2.72 |  |  |  |  |  |
| PELV | 0.558 | 4.03 | 2.57 | 3.78 |  |  |  |  |
| HUM | -0.115 | 3.95 | 1.85 | 3.00 | 4.28 |  |  |  |
| UL | 0.118 | 5.96 | 2.10 | 3.77 | 6.02 | 9.59 |  |  |
| FEM | 0.283 | 3.53 | 0.511 | 1.11 | 2.94 | 5.88 | 5.87 |  |
| TIB | 0.168 | 4.71 | 0.966 | 1.62 | 4.37 | 8.52 | 7.73 | 10.77 |

Eigenvalues and eigenvectors of **D**

|  | **d**_1_ | **d**_2_ | **d**_3_ | **d**_4_ | **d**_5_ | **d**_6_ |
| --- | --- | --- | --- | --- | --- | --- |
| *λ* (× 10^-3^) | 31.9 | 8.03 | 2.00 | 1.01 | 0.31 | 0.17 |
| % var | 73.46 | 18.50 | 4.61 | 2.32 | 0.72 | 0.39 |
| JL | 0.017 | 0.035 | 0.261 | 0.651 | 0.321 | -0.104 |
| HW | 0.365 | 0.369 | 0.399 | 0.072 | -0.604 | -0.292 |
| PECT | 0.140 | 0.424 | 0.342 | -0.621 | 0.306 | 0.007 |
| PELV | 0.216 | 0.511 | 0.073 | 0.348 | 0.379 | 0.311 |
| HUM | 0.323 | 0.209 | -0.521 | -0.089 | -0.063 | 0.503 |
| UL | 0.532 | 0.039 | -0.494 | 0.113 | -0.008 | -0.477 |
| FEM | 0.373 | -0.375 | 0.313 | 0.104 | -0.310 | 0.553 |
| TIB | 0.522 | -0.485 | 0.192 | -0.183 | 0.442 | -0.144 |

Species coordinates within first two divergence eigenvectors

|  | **d**_1_ | **d**_2_ |
| --- | --- | --- |
| CRIS | 0.206 | 0.011 |
| EVER | 0.125 | 0.030 |
| GRAH | 0.024 | 0.080 |
| LINE | 0.112 | -0.075 |
| PULC | -0.189 | -0.157 |
| SAGR | 0.013 | 0.009 |
| SMAR | -0.291 | 0.102 |

Variance-covariance matrix of phylogenetically independent contrasts (**D**_IC_, × 10^-4^)

|  | JL | HW | PECT | PELV | HUM | UL | FEM | TIB |
| --- | --- | --- | --- | --- | --- | --- | --- | --- |
| JL | 0.138 |  |  |  |  |  |  |  |
| HW | -0.064 | 1.08 |  |  |  |  |  |  |
| PECT | -0.009 | 0.868 | 0.825 |  |  |  |  |  |
| PELV | 0.015 | 0.696 | 0.645 | 0.677 |  |  |  |  |
| HUM | -0.268 | 0.502 | 0.350 | 0.393 | 0.830 |  |  |  |
| UL | -0.349 | 0.631 | 0.384 | 0.348 | 0.994 | 1.39 |  |  |
| FEM | -0.172 | 0.186 | 0.114 | -0.177 | 0.149 | 0.357 | 0.570 |  |
| TIB | -0.283 | 0.282 | 0.197 | -0.183 | 0.403 | 0.832 | 0.790 | 1.31 |

Eigenvalues and eigenvectors of **D**_IC_

|  | **d**_IC1_ | **d**_IC2_ | **d**_IC3_ | **d**_IC4_ | **d**_IC5_ |
| --- | --- | --- | --- | --- | --- |
| *λ* (× 10^-4^) | 3.79 | 1.95 | 0.88 | 0.099 | 0.087 |
| % var | 55.65 | 28.71 | 12.91 | 1.45 | 1.28 |
| JL | -0.128 | 0.141 | 0.141 | 0.242 | 0.405 |
| HW | 0.423 | 0.367 | 0.324 | -0.572 | 0.363 |
| PECT | 0.327 | 0.369 | 0.390 | 0.377 | -0.287 |
| PELV | 0.239 | 0.480 | -0.033 | 0.308 | -0.128 |
| HUM | 0.399 | 0.016 | -0.482 | -0.090 | -0.511 |
| UL | 0.544 | -0.164 | -0.471 | 0.048 | 0.466 |
| FEM | 0.202 | -0.363 | 0.386 | -0.413 | -0.341 |
| TIB | 0.382 | -0.566 | 0.346 | 0.443 | 0.109 |

Evolutionary rate matrix (**R**, × 10^-4^)

|  | JL | HW | PECT | PELV | HUM | UL | FEM | TIB |
| --- | --- | --- | --- | --- | --- | --- | --- | --- |
| JL | 0.148 |  |  |  |  |  |  |  |
| HW | 0.179 | 1.92 |  |  |  |  |  |  |
| PECT | 0.042 | 0.999 | 0.786 |  |  |  |  |  |
| PELV | 0.177 | 1.45 | 0.892 | 1.33 |  |  |  |  |
| HUM | 0.007 | 1.39 | 0.702 | 1.10 | 1.34 |  |  |  |
| UL | 0.063 | 1.94 | 0.804 | 1.36 | 1.80 | 2.68 |  |  |
| FEM | 0.096 | 1.06 | 0.252 | 0.450 | 0.787 | 1.44 | 1.39 |  |
| TIB | 0.065 | 1.34 | 0.376 | 0.597 | 1.12 | 2.02 | 1.73 | 2.32 |

Eigenvalues and eigenvectors of **R**

|  | **r**_1_ | **r**_2_ | **r**_3_ | **r**_4_ | **r**_5_ | **r**_6_ |
| --- | --- | --- | --- | --- | --- | --- |
| *λ* (× 10^-4^) | 9.03 | 2.04 | 0.51 | 0.21 | 0.077 | 0.034 |
| % var | 75.85 | 17.17 | 4.25 | 1.81 | 0.649 | 0.282 |
| JL | 0.026 | 0.037 | 0.320 | 0.592 | -0.389 | -0.033 |
| HW | 0.430 | 0.269 | 0.374 | 0.100 | 0.534 | -0.437 |
| PECT | 0.196 | 0.377 | 0.298 | -0.686 | -0.234 | -0.020 |
| PELV | 0.300 | 0.483 | 0.160 | 0.271 | -0.338 | 0.372 |
| HUM | 0.356 | 0.178 | -0.475 | -0.068 | 0.156 | 0.522 |
| UL | 0.528 | -0.034 | -0.525 | 0.175 | -0.103 | -0.413 |
| FEM | 0.317 | -0.442 | 0.348 | 0.079 | 0.384 | 0.468 |
| TIB | 0.424 | -0.567 | 0.143 | -0.233 | -0.460 | -0.082 |

Table S3. Divergence matrix for 15 *Anolis* species using traits measured in wild-caught adult males. Sample sizes are smaller for species from Jamaica because of permitting restrictions.

Sampling information

| Species | Code | Ecomorph | Island | Location | n |
| --- | --- | --- | --- | --- | --- |
| *A. angusticeps* | ANGU | twig | South Bimini | 25.70°N, 79.28°W | 79 |
| *A. coelestinus* | COEL | trunk-crown | Hispaniola | 18.06°N, 71.11°W | 100 |
| *A. cristatellus* | CRIS | trunk-ground | Puerto Rico | 18.05°N, 65.83°W | 99 |
| *A. cybotes* | CYBO | trunk-ground | Hispaniola | 18.06°N, 71.11°W | 100 |
| *A. distichus* | DIST | trunk | South Bimini | 25.70°N, 79.28°W | 97 |
| *A. evermanni* | EVER | trunk-crown | Puerto Rico | 18.27°N, 65.72°W | 92 |
| *A. garmani* | GARM | crown giant | Jamaica | 18.32°N, 76.81°W | 15 |
| *A. grahami* | GRAH | trunk-crown | Jamaica | 18.32°N, 76.81°W | 5 |
| *A. gundlachi* | GUND | trunk-ground | Puerto Rico | 18.11°N, 66.05°W | 106 |
| *A. krugi* | KRUG | grass-bush | Puerto Rico | 18.26°N, 65.71°W | 114 |
| *A. lineatopus* | LINE | trunk-ground | Jamaica | 18.32°N, 76.81°W | 19 |
| *A. olssoni* | OLSS | grass-bush | Hispaniola | 18.06°N, 71.11°W | 102 |
| *A. pulchellus* | PULC | grass-bush | Puerto Rico | 18.26°N, 65.71°W | 102 |
| *A. sagrei* | SAGR | trunk-ground | South Bimini | 25.70°N, 79.28°W | 160 |
| *A. smaragdinus* | SMAR | trunk-crown | South Bimini | 25.70°N, 79.28°W | 60 |

Back-transformed species means (mm)

|  | Species | | | | | | | |
| --- | --- | --- | --- | --- | --- | --- | --- | --- |
| Trait | ANGU | COEL | CRIS | CYBO | DIST | EVER | GARM | GRAH |
| JL | 17.825 | 16.767 | 16.470 | 17.946 | 14.909 | 16.244 | 17.257 | 16.902 |
| HW | 8.919 | 9.344 | 10.933 | 12.082 | 8.703 | 9.373 | 9.004 | 10.956 |
| PECT | 6.406 | 7.842 | 8.777 | 7.501 | 6.880 | 8.229 | 7.521 | 8.508 |
| PELV | 5.111 | 6.005 | 6.505 | 6.395 | 6.935 | 6.415 | 4.841 | 6.581 |
| HUM | 6.484 | 8.054 | 9.199 | 9.215 | 9.346 | 9.382 | 8.105 | 8.047 |
| UL | 5.316 | 6.438 | 7.685 | 8.078 | 8.221 | 7.914 | 6.926 | 6.890 |
| FEM | 8.542 | 10.916 | 13.784 | 13.946 | 11.729 | 12.313 | 12.233 | 11.815 |
| TIB | 7.607 | 9.676 | 12.202 | 12.567 | 10.826 | 11.375 | 10.450 | 10.727 |
|  |  | | | | | | |  |
| Trait | GUND | KRUG | LINE | OLSS | PULC | SAGR | SMAR |  |
| JL | 16.811 | 16.363 | 17.969 | 14.675 | 17.677 | 15.747 | 17.626 |  |
| HW | 10.660 | 10.380 | 10.448 | 6.331 | 9.156 | 9.082 | 9.542 |  |
| PECT | 8.292 | 7.562 | 7.058 | 5.152 | 6.809 | 7.628 | 7.143 |  |
| PELV | 6.043 | 5.149 | 6.073 | 4.168 | 5.011 | 5.792 | 5.753 |  |
| HUM | 9.739 | 8.173 | 8.063 | 7.057 | 7.361 | 7.794 | 6.983 |  |
| UL | 7.972 | 6.785 | 6.862 | 5.367 | 6.187 | 6.451 | 5.388 |  |
| FEM | 14.950 | 12.499 | 12.836 | 11.286 | 11.178 | 11.555 | 9.886 |  |
| TIB | 13.229 | 11.332 | 11.773 | 10.281 | 9.941 | 10.828 | 8.639 |  |

Variance-covariance matrix of species means (**D**_15_, × 10^-3^)

|  | JL | HW | PECT | PELV | HUM | UL | FEM | TIB |
| --- | --- | --- | --- | --- | --- | --- | --- | --- |
| JL | 4.04 |  |  |  |  |  |  |  |
| HW | 5.75 | 22.17 |  |  |  |  |  |  |
| PECT | 2.31 | 15.64 | 17.52 |  |  |  |  |  |
| PELV | 0.722 | 14.07 | 13.19 | 19.80 |  |  |  |  |
| HUM | -1.44 | 9.23 | 10.23 | 11.62 | 14.99 |  |  |  |
| UL | -0.924 | 12.69 | 12.67 | 14.84 | 17.87 | 22.46 |  |  |
| FEM | -0.711 | 10.14 | 9.10 | 7.21 | 14.34 | 16.73 | 19.17 |  |
| TIB | -1.47 | 9.75 | 8.76 | 8.19 | 14.85 | 17.44 | 19.44 | 20.31 |

Eigenvalues and eigenvectors of **D**_15_

|  | **d**_15,1_ | **d**_15,2_ | **d**_15,3_ | **d**_15,4_ | **d**_15,5_ | **d**_15,6_ | **d**_15,7_ | **d**_15,8_ |
| --- | --- | --- | --- | --- | --- | --- | --- | --- |
| *λ* (× 10^-3^) | 96.9 | 24.6 | 11.0 | 4.27 | 2.57 | 0.70 | 0.31 | 0.093 |
| % var | 69.98 | 17.49 | 7.87 | 3.04 | 1.83 | 0.49 | 0.22 | 0.06 |
| JL | 0.014 | 0.262 | 0.356 | 0.240 | 0.325 | 0.766 | 0.146 | 0.183 |
| HW | 0.361 | 0.511 | 0.474 | 0.324 | 0.050 | -0.488 | -0.194 | 0.004 |
| PECT | 0.335 | 0.380 | 0.067 | -0.841 | -0.068 | 0.092 | 0.136 | 0.025 |
| PELV | 0.343 | 0.382 | -0.581 | 0.331 | -0.467 | 0.243 | 0.041 | -0.100 |
| HUM | 0.368 | -0.184 | -0.242 | -0.072 | 0.284 | 0.117 | -0.725 | 0.384 |
| UL | 0.453 | -0.150 | -0.286 | 0.092 | 0.629 | -0.163 | 0.472 | -0.192 |
| FEM | 0.381 | -0.385 | 0.333 | -0.006 | -0.195 | 0.255 | -0.209 | -0.669 |
| TIB | 0.392 | -0.419 | 0.235 | 0.089 | -0.393 | -0.038 | 0.359 | 0.568 |

**Table S4.** Krzanowski’s common subspace analysis. Axes of conserved genetic variance across 7 *Anolis* species. The value of *p* indicates the number of species that putatively share each axis. *θ* indicates the angle (in degrees) between **h** and the subspace defined by the first four eigenvectors of the **G** matrix of each species (abbreviated with a four-letter code). *θ_h,g_* indicates the angle (in degrees) between each **h** and the eigenvector from Table S1 with the same index (e.g., **h**_2_ and **g**_2_). Percent variance is calculated by projecting each **h** through each **G** using **h**^T^**Gh** and dividing by the total genetic variance. *θ_h,d_* values represent angles between each **h** and the first two axes of divergence (Table S2).

|  | **h**_1_ | **h**_2_ | **h**_3_ | **h**_4_ |
| --- | --- | --- | --- | --- |
| *p* | 6.94 | 6.56 | 5.89 | 4.32 |
| JL | 0.187 | 0.185 | 0.204 | -0.079 |
| HW | 0.139 | 0.391 | 0.068 | -0.348 |
| PECT | 0.250 | 0.756 | 0.331 | 0.186 |
| PELV | 0.197 | 0.320 | -0.910 | 0.128 |
| HUM | 0.500 | -0.180 | 0.014 | -0.380 |
| UL | 0.491 | -0.186 | -0.051 | -0.457 |
| FEM | 0.401 | -0.172 | 0.071 | 0.435 |
| TIB | 0.438 | -0.207 | 0.088 | 0.527 |
|  |  |  |  |  |
| *θ* |  |  |  |  |
| CRIS | 6.02 | 7.62 | 40.60 | 52.17 |
| EVER | 3.88 | 10.77 | 8.34 | 30.05 |
| GRAH | 3.53 | 18.19 | 18.01 | 45.25 |
| LINE | 5.46 | 16.55 | 20.58 | 7.98 |
| PULC | 5.79 | 10.42 | 18.36 | 22.19 |
| SAGR | 3.64 | 15.44 | 22.39 | 70.64 |
| SMAR | 8.60 | 19.38 | 26.62 | 29.89 |
|  |  |  |  |  |
| *θ_h,g_* |  |  |  |  |
| CRIS | 30.67 | 36.19 | 57.69 | 55.10 |
| EVER | 19.20 | 20.09 | 67.15 | 73.31 |
| GRAH | 10.63 | 40.39 | 59.38 | 67.10 |
| LINE | 11.20 | 53.05 | 61.23 | 81.95 |
| PULC | 11.37 | 29.10 | 56.29 | 53.52 |
| SAGR | 25.78 | 76.56 | 70.48 | 71.50 |
| SMAR | 18.69 | 35.12 | 42.16 | 40.52 |

| % var |  |  |  |  |
| --- | --- | --- | --- | --- |
| CRIS | 46.4 | 29.6 | 5.5 | 4.8 |
| EVER | 57.4 | 21.6 | 5.2 | 7.8 |
| GRAH | 60.8 | 13.6 | 8.8 | 5.2 |
| LINE | 54.8 | 13.0 | 10.4 | 15.4 |
| PULC | 50.0 | 21.2 | 10.6 | 10.7 |
| SAGR | 47.2 | 19.4 | 13.5 | 3.1 |
| SMAR | 52.9 | 18.9 | 8.2 | 7.0 |
|  |  |  |  |  |
| *θ_h,d1_* | 21.03 | 89.49 | 85.87 | 89.83 |
| *θ_h,d2_* | 88.56 | 40.97 | 68.83 | 59.77 |

**Table S5.** Genetic covariance tensor analysis. **G** varied across species in six subspaces (eigentensors, **E**_1_-**E­**_6_). For each, percent divergence explained and coordinates of each species within the eigentensor are shown (Hine et al. 2009). Similar coordinate values indicate that a pair of species shows similarity in **G** within a particular subspace. The largest ten eigenvectors (in terms of percent divergence in **G** explained) of these subspaces are shown as **e***_ij_*, indexed by subspace (*i*) and eigenvector number within each subspace (*j*). Because eigenvalues can be negative, the 8^th^ eigenvalue sometimes explains the most variance. Angles (in degrees) are shown between each of these vectors and axes of both shared genetic variance (**h**, Table S4) and divergence (**d**, Table S2).

Percent variance explained and species coordinates within eigentensor (× 10^-3^)

|  | **E**_1_ | **E**_2_ | **E**_3_ | **E**_4_ | **E**_5_ | **E**_6_ |
| --- | --- | --- | --- | --- | --- | --- |
| % var | 48.09 | 23.90 | 12.47 | 8.24 | 4.44 | 2.85 |
| CRIS | 3.92 | 0.007 | -1.168 | 0.078 | 0.903 | 0.232 |
| EVER | 4.44 | -1.11 | 0.428 | -0.150 | 0.463 | 0.230 |
| GRAH | 2.57 | -0.906 | -0.436 | -1.012 | 0.284 | -0.338 |
| LINE | 2.81 | -2.213 | -0.965 | -0.179 | 0.671 | 0.231 |
| PULC | 2.48 | -1.08 | -0.556 | -0.180 | 0.947 | 0.176 |
| SAGR | 1.80 | -0.067 | -0.242 | -0.392 | 0.090 | 0.424 |
| SMAR | 1.54 | -0.990 | -0.089 | 0.409 | 0.366 | 0.431 |

First ten eigenvectors of eigentensors

|  | **e**_11_ | **e**_28_ | **e**_12_ | **e**_21_ | **e**_38_ | **e**_31_ | **e**_41_ | **e**_27_ | **e**_48_ | **e**_51_ |
| --- | --- | --- | --- | --- | --- | --- | --- | --- | --- | --- |
| % var | 40.7 | 12.7 | 6.76 | 6.72 | 6.46 | 4.38 | 4.28 | 2.69 | 2.43 | 2.14 |
| JL | -0.020 | -0.349 | -0.219 | -0.146 | 0.168 | 0.209 | -0.019 | -0.234 | -0.256 | 0.242 |
| HW | 0.006 | -0.182 | 0.389 | 0.264 | 0.189 | 0.124 | 0.024 | -0.226 | -0.166 | 0.223 |
| PECT | -0.104 | -0.512 | 0.796 | 0.651 | -0.184 | -0.036 | 0.909 | -0.164 | 0.041 | 0.217 |
| PELV | -0.019 | -0.315 | 0.308 | 0.125 | -0.346 | -0.332 | 0.344 | 0.446 | 0.044 | 0.594 |
| HUM | 0.459 | -0.110 | -0.143 | -0.463 | 0.701 | -0.034 | -0.210 | -0.318 | 0.565 | 0.464 |
| UL | 0.411 | -0.216 | -0.038 | -0.168 | 0.523 | -0.269 | 0.094 | -0.649 | 0.708 | 0.425 |
| FEM | 0.531 | -0.512 | 0.202 | -0.219 | 0.126 | -0.385 | 0.029 | 0.232 | 0.141 | -0.010 |
| TIB | 0.571 | -0.403 | 0.098 | -0.423 | -0.042 | -0.779 | -0.024 | 0.304 | 0.250 | 0.311 |
|  |  |  |  |  |  |  |  |  |  |  |
| *θ_e,h1_* | **30.40** | **34.49** | 72.12 | 67.06 | 54.38 | 48.42 | 76.24 | 73.77 | **41.91** | **34.86** |
| *θ_e,h2_* | 62.95 | 66.93 | **37.88** | **31.98** | 67.62 | 76.25 | **34.64** | 87.81 | 67.88 | 74.91 |
| *θ_e,d1_* | **32.09** | **40.15** | 67.88 | 70.78 | 59.92 | 42.85 | 79.01 | 77.54 | 46.15 | **38.81** |
| *θ_e,d2_* | 65.39 | 84.18 | 61.58 | 51.93 | 87.77 | 68.09 | 58.00 | 74.93 | 86.63 | 63.12 |

**Table S6.** Tests from Table 4 using alternative estimates of species divergence (Table S3). Statistical significance of alignment was assessed by comparison to a null distribution of randomly generated pairs of vectors (see Methods); * *P* < 0.05, ** *P* < 0.01, ***** *P* < 0.001.

|  | **d**_IC1_ | **d**_IC2_ | **r**_1_ | **r**_2_ | **d**_15,1_ | **d**_15,2_ |
| --- | --- | --- | --- | --- | --- | --- |
| **h**_1_ | 28.5** | 78.4 | 22.5*** | 83.1 | 20.7*** | 77.7 |
| **h**_2_ | 79.7 | 36.0** | 83.6 | 44.0* | 77.9 | 29.4** |
| **e**_11_ | 45.6* | 51.4 | 39.8* | 57.4 | 42.1* | 50.3 |
| **e**_28_ | 46.1* | 89.5 | 39.8* | 88.0 | 32.6** | 85.4 |

**Figure S1**. Species means of wild-caught adult males along the first two divergence axes (Table S3). Species abbreviations are as in Table S3. Ecomorphs are shown using two-letter codes (CG = crown giant, GB = grass-bush, TC = trunk-crown, TG = trunk-ground, TR = trunk, TW = twig).

**Figure S2.** Relationship between log-transformed genetic variance (*G*) and four different estimates of divergence in a set of eight orthogonal trait combinations. Points using the divergence matrix (**D**) are replotted from Fig. 4 and shown in black. Purple points use an alternative divergence matrix calculated from phenotypic data from wild-caught adult males (**D**_15_). Points in blue and green correct for phylogenetic relationships in slightly different ways (evolutionary rate, **R**, and divergence of independent contrasts, **D**_IC_, respectively). Trait combinations are defined in a subspace common to particular divergence (or rate) matrix and the estimated ancestral **G** matrix (**G**­_anc_).
